# The shadowing effect of initial expectation on learning asymmetry

**DOI:** 10.1371/journal.pcbi.1010751

**Published:** 2023-07-24

**Authors:** Yinmei Ni, Jingwei Sun, Jian Li

**Affiliations:** 1 School of Psychological and Cognitive Sciences and Beijing Key Laboratory of Behavior and Mental Health, Peking University, Beijing, China; 2 PKU-IDG/McGovern Institute for Brain Research, Peking University, Beijing, China; 3 Lenovo Research, Lenovo Group, Beijing, China; McGill University, CANADA

## Abstract

Evidence for positivity and optimism bias abounds in high-level belief updates. However, no consensus has been reached regarding whether learning asymmetries exist in more elementary forms of updates such as reinforcement learning (RL). In RL, the learning asymmetry concerns the sensitivity difference in incorporating positive and negative prediction errors (PE) into value estimation, namely the asymmetry of learning rates associated with positive and negative PEs. Although RL has been established as a canonical framework in characterizing interactions between agent and environment, the direction of learning asymmetry remains controversial. Here, we propose that part of the controversy stems from the fact that people may have different value expectations before entering the learning environment. Such a default value expectation influences how PEs are calculated and consequently biases subjects’ choices. We test this hypothesis in two learning experiments with stable or varying reinforcement probabilities, across monetary gains, losses, and gain-loss mixed environments. Our results consistently support the model incorporating both asymmetric learning rates and the initial value expectation, highlighting the role of initial expectation in value updating and choice preference. Further simulation and model parameter recovery analyses confirm the unique contribution of initial value expectation in accessing learning rate asymmetry.

## Author summary

While Reinforcement learning models have long been applied to characterize learning behavior, where value update stands at the core of the learning process, it remains controversial whether and how learning is biased when updating from positive and negative PEs. Here, through model comparison, simulation, and recovery analyses, we show that accurate identification of learning asymmetry is contingent on taking into account of subjects’ default value expectation in both monetary gain and loss environments. Our results stress the importance of initial expectation specification, especially in studies investigating learning asymmetry.

## Introduction

During the interaction with an uncertain environment, humans learn by trial-and-error, incorporating feedbacks into existing beliefs to accrue reward and avoid punishment, as the reinforcement learning (RL) theory prescribes [1]. When an action leads to better-than-expected outcomes and generates a positive prediction error, such an action tends to be repeated. In contrast, if an action is followed by a worse-than-expected outcome (negative prediction error), the tendency to repeat that action is reduced. Early RL models typically assume that people’s sensitivities (learning rates) towards positive and negative prediction errors are the same (symmetric) [1–3]. Recently, however, evidence starts to emerge that the impacts of positive and negative outcomes might be different [4–9], and distinct neural circuits may subserve learning from positive and negative prediction errors[10,11].

Surprisingly, no consensus has been reached regarding the direction of learning asymmetry. In the cases of high-level and ego-related belief updates, it has been shown that people tend to overestimate the likelihood of positive events and underestimate the likelihood of negative ones, a bias termed unrealistic optimism, in order to maintain the self-serving psychological status [12–16]. For example, when faced with new information about adverse life events, participants update their beliefs in response to desirable information (better than expected) more than to undesirable information (worse than expected) [5,17–18] (but also see [19,20]). However, results for the learning asymmetry in more elementary forms of updates such as RL are rather mixed. While some studies using standard RL paradigms found that humans’ positive learning rates were larger than the negative ones, yielding an optimistic RL bias [4,16,21]. Other studies, however, obtained opposite results with larger negative learning rates [6,7,22], consistent with the prevalent psychological phenomenon “bad is stronger than good” [23].

We hypothesize that part of the discrepancies in the previous literature stems from the often less appreciated fact that the initial or default value expectation (*Q*_0_ in a Q-learning framework) plays a critical role in identifying the direction of learning asymmetry. In a standard two-arm bandit RL paradigm, the action value is updated by the product of learning rate (α) and PE (δ_*t*_), which is the difference between the obtained reward (*R*_*t*_) and action value (*Q*_*t*−1_) of the previous trial for a particular trial *t*. Intuitively, setting the initial action value *Q*_0_ would have a direct impact on the calculation of PE [24]. For example, under the circumstance where the endowed initial action value is higher than the true value of the selected option, the original positive prediction error is then down-scaled and the negative one up-scaled, assuming a binary reinforcement structure. The distortion of the PEs can only be balanced by scaling the learning rates in the opposite directions to achieve the same amount of action value update (α⋅δ) as it should have been under the RL framework. It, therefore, creates an ostensibly positive bias in learning rates (the positive learning rate appears to be larger than the negative one). On the contrary, a negative bias in learning rates can emerge if the initial action value is mis-specified to be lower than the true action value. However, a majority of recent studies focused exclusively on the role of learning rates in characterizing participants’ learning behavior and considered *Q*_0_ as a mundane initialization parameter, due to the belief that the impact of initial expectations would be “washed out” after enough trials of learning. Indeed, while some studies initialized *Q*_0_ to zero, probably reflecting the fact that participants possessed no information about options before the task [6–8,21,25]; other studies adopted the median or mean values of the possible option outcomes as *Q*_0_, corresponding to an *a priori* expectation of receiving different outcomes with equal probabilities [4,26–28]. Only a limited number of studies tested the models where *Q*_0_ was treated as a free parameter [29,30].

However, it is possible that there are significant individual differences in the initial expectations. Such initial expectations could reflect the internal motivation, or response vigor that participants carry into the task [31,32]. In addition, the initial expectation might be susceptible to instructions or context cues, which can have significant impacts on participants’ choice behavior [29,32–34]. Furthermore, contrary to the standard view, the initial value expectation may have long-lasting effects on subsequent choice behavior due to the intricate interplay between choice selection and the update of action value. For example, if the interaction with a specific option widens the action value gap (between options) due to the initial action value (*Q*_0_) specification, then the lower valued option is less likely to be selected, making it harder to learn the true value of that option [6]. Therefore, RL models that do not take initial expectations into account may risk attributing the variance in choice behavior to the wrong cause by biasing the estimation of the underlying learning rates.

To test this hypothesis, we conducted two experiments where subjects were asked to choose between two partially reinforced options in both the stable (fixed probability, Experiment 1) and varying (random-walk, Experiment 2) probability environments. Two groups of subjects repeatedly chose from pairs of options with probabilistic binary reward outcomes to earn monetary rewards, avoid losses, or both. We tested different variants of RL models against participants’ behavior, with the focus on learning asymmetry and initial expectations. Model comparison results showed that the RL model with asymmetric learning rates and individualized initial expectations performed best in explaining participants’ behavioral data in both Experiments 1 & 2. Further simulation and recovery analyses confirmed our results and demonstrated the characteristic impacts on learning asymmetry by omitting the initial expectation.

## Results

### Logistic regression and computational models

**Fig 1:**
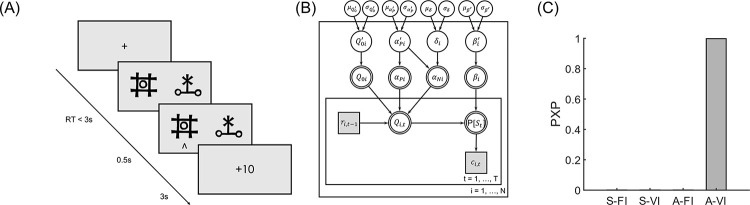
Experimental design and computational model of Experiment 1 (stable probability). (A) The trial procedure of experiment 1. In each trial, participants chose between two options, each of which was partially reinforced with a fixed probability. (B) The hierarchical Bayesian modeling procedure assumes that the parameter set {*Q*_0_, *α*_*P*_, *α*_*N*_, *β*} for participant *i* (*i*∈{1,…,*N*}) was Φ transformed from $\{Q_{0}^{\prime },\alpha_{P}^{\prime },\delta ,\beta^{\prime }\}$, which is, in turn, a sample drawn from the hyper distributions (see Methods). *c*_*i*,*t*_, *r*_*i*,*t*_ are participant *i*’s choice and the reward received at trial *t* (*t*∈{1,…,*T*}). (C) Model comparison results. The deviance information criterion (DIC) for each candidate model was used in the Bayesian model comparison to generate the protected exceedance probability (PXP), an index indicating the probability that a specific model is the best among the candidate models. For all the models considered, model A-VI outperformed other models with PXP > 0.99.

Twenty-eight subjects (one subject excluded due to technical problems) participated in Experiment 1, where they were asked to choose from pairs of options (denoted with different visual stimuli) that were partially reinforced with fixed probabilities (Fig 1A). Experiment 1 consisted of two conditions (monetary gain and loss) and each condition consisted of four pairs of options and their probabilities for winning (Gain) or losing (Loss) were 40–60%, 25–75%, 25–25%, and 75–75%, respectively. Each pair of options was grouped into a mini-block and consisted of 32 trials.

The mixed-effect logistic regression analysis (lme4 package in R v4.2.2 [35]) showed that subjects’ choices were sensitive to the past reward history (last trial outcome on the stay probability: *β* = 0.958, *p* < 0.001), indicating that subjects did pay attention to the tasks and learned by trial-and-error. Across trials, the proportion of correct choices increased in both the Gain and Loss conditions, with higher correct choice rates in the 25–75% than in the 40–60% block (*p-values* < 0.001, S1A and S1B Fig). To test our hypothesis concerning learning asymmetry and the initial expectation, we fitted the data with a standard Q-learning model assuming different learning rates for positive and negative prediction errors with idiosyncratic initial expectations (model A-VI). We also fitted three variants of this model, one with asymmetric learning rates and fixed initial expectations (A-FI, the initial expectations were 0.5, -0.5, and 0 in the Gain, Loss, and Mixed conditions, respectively, see S1 Table for model details), another with symmetric learning rates and individualized initial expectations (S-VI), and lastly the one with symmetric learning rates and fixed initial expectations and (S-FI). We used a Bayesian hierarchical modeling procedure to fit the data (Fig 1B and see Methods). Deviance information criterion (DIC) was then used to perform the Bayesian model selection among the candidate models. The protected exceedance probability (PXP, a model comparison index of the probability that a particular model is more frequent in the population than all other models under consideration [36,37]) indicated that the A-VI model performed the best in explaining subjects’ choice behavior (Fig 1C, and S1 and S2 Tables). Similar model comparison results were obtained when the A-FI and S-FI models were instead endowed with initial expectations (*Q*_0_) of 0 (S1 Table).

### Learning asymmetry revealed by the inclusion of initial expectation

**Fig 2:**
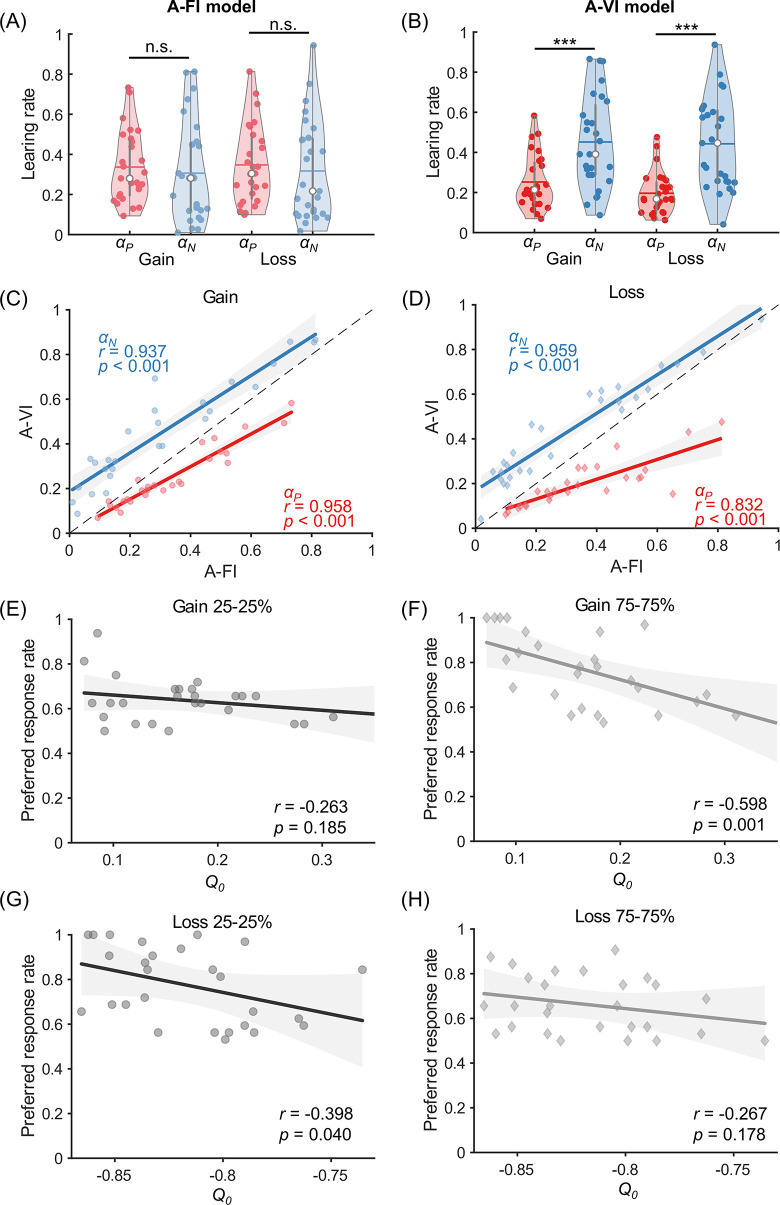
Model fitting results of Experiment 1. (A-B) Estimated learning rates for the Gain and Loss conditions by the A-FI (A) and A-VI models (B). The positive (α_*P*_) and negative (α_*N*_) learning rates are denoted in red and blue, and the empty circle and the vertical line denote the median and the mean of the learning rates across subjects. (C-D) Significant correlations of α_*P*_ and α_*N*_ were observed between A-FI and A-VI. models in both the Gain (C) and Loss (D) conditions. (E) No significant correlation between the preferred response rate (PRR) and *Q*_0_ in the 25–25% block of the Gain condition. (F) However, a strong correlation was observed in the 75–75% block of the Gain condition where there was a significant mismatch between the individual and the true *Q*_0_ (0.75) of the block. (G-H) Similar patterns of correlational results between *Q*_0_ and PRR in the 25–25% (G) and 75–75% (H) blocks of the Loss condition. Asterisks (***) denote *p* < 0.001 (paired t-test) and n.s. stands for no statistical significance (*p* > 0.05).

Since most previous literature investigating learning asymmetry did not consider the possibility that the initial expectation may vary across subjects, we specifically examined the difference of learning rates estimated from the A-VI and A-FI models. We found the directions of learning asymmetry suggested by these two models were different. The positive and negative learning rates were not significantly different according to the A-FI model in the two conditions (Fig 2A, *p* = 0.265 and *p* = 0.506 for the Gain and Loss conditions, respectively, paired t-test). However, after incorporating the initial expectation (A-VI model), a negative learning asymmetry was revealed in both the Gain and the Loss (Fig 2B, *p-values* < 0.001, paired t-test) conditions. Importantly, there was no significant correlation between learning rates and initial expectation (*Q*_0_) in either the Gain or Loss condition (A-VI model), suggesting independent contributions of *Q*_0_ and learning rates in explaining choice differences across subjects (S2 Fig; *r* = -0.120, *p* = 0.550 between *Q*_0_ & *α*_*P*_; *r* = 0.235, *p* = 0.237 between *Q*_0_ & *α*_*N*_ in the Gain condition; *r* = 0.017, *p* = 0.935 between *Q*_0_ & *α*_*P*_, *r* = 0.362, *p* = 0.064 between *Q*_0_ & *α*_*N*_, in the Loss condition).

Despite the learning asymmetry reversal by considering individual *Q*_0_ in the A-VI model, however, closer examination of the learning rates estimated from the A-VI and A-FI models showed interesting correlations. Indeed, *α*_*P*_ and *α*_*N*_ were strongly correlated with their counterparts between the two models for both the Gain (*α*_*P*_: *r* = 0.958, *p* < 0.001; *α*_*N*_: *r* = 0.937, *p* < 0.001; Fig 2C) and Loss conditions (*α*_*P*_: *r* = 0.832, *p* < 0.001; *α*_*N*_: *r* = 0.959, *p* < 0.001; Fig 2D), suggesting the relative rank of the individual difference in learning rates (positive or negative) is well preserved in both A-VI and A-FI models.

In Experiment 1, we also included 25–25% and 75–75% blocks which according to the previous literature might provide crucial evidence to support the optimistic RL hypothesis, as opposed to the symmetric learning alternative [4,24,28]. We also tested such hypotheses and found that the ‘preferred response’ rate (PRR), a term defined as the choice rate of the option most frequently chosen by the subject and potentially reflecting the tendency to overestimate certain option value, was correlated with *Q*_0_. More specifically, PRR was only negatively correlated with *Q*_0_ in the 75–75% Gain condition (*r* = -0.598, *p* = 0.001; Fig 2F) and 25–25% Loss condition (*r* = -0.398, *p* = 0.04; Fig 2G) where there was a considerable mismatch between participants’ mean *Q*_0_ (mean±s.d.: 0.171±0.074 and -0.817±0.034 in the Gain and Loss conditions) and the true action value (0.75 in the 75–75% Gain block and -0.25 in the 25–25% Loss block, respectively), indicating that the PRRs might instead be driven by the rather inaccurate initial expectations. Indeed, when the initial expectation was close to the true option values (25–25% Gain and 75–75% Loss), such correlation was not observed (Fig 2E, *r* = -0.263, *p* = 0.185 in the 25–25% Gain; Fig 2H, *r* = -0.267, *p* = 0.178 in the 75–75% Loss). These results suggest that the magnitudes of the discrepancies between the individual and true *Q*_0_ may underlie the associations between individual *Q*_0_ and PRR (Fig 2E–2H).

### Model simulation and parameter recovery

To comprehensively investigate the influence of initial expectations on the estimation of learning rates, we further performed a model simulation analysis. We systematically varied the levels of the initial expectation (*Q*_0_ = 0, 0.25, 0.5, 0.75, 1) as well as the asymmetry of the positive and negative learning rates ((*α*_*P*_, *α*_*N*_) = (0.1, 0.7), (0.2, 0.6), (0.3, 0.5), (0.4, 0.4), (0.5, 0.3), (0.6, 0.2), (0.7, 0.1)) to simulate datasets using the A-VI model. Each combination of parameters generated 30 datasets with each dataset consisting of 30 hypothetical subjects, resulting in 1050 (35 x 30) datasets in total. We then applied the same model fitting procedure with A-VI and A-FI models to the simulated datasets.

**Fig 3:**
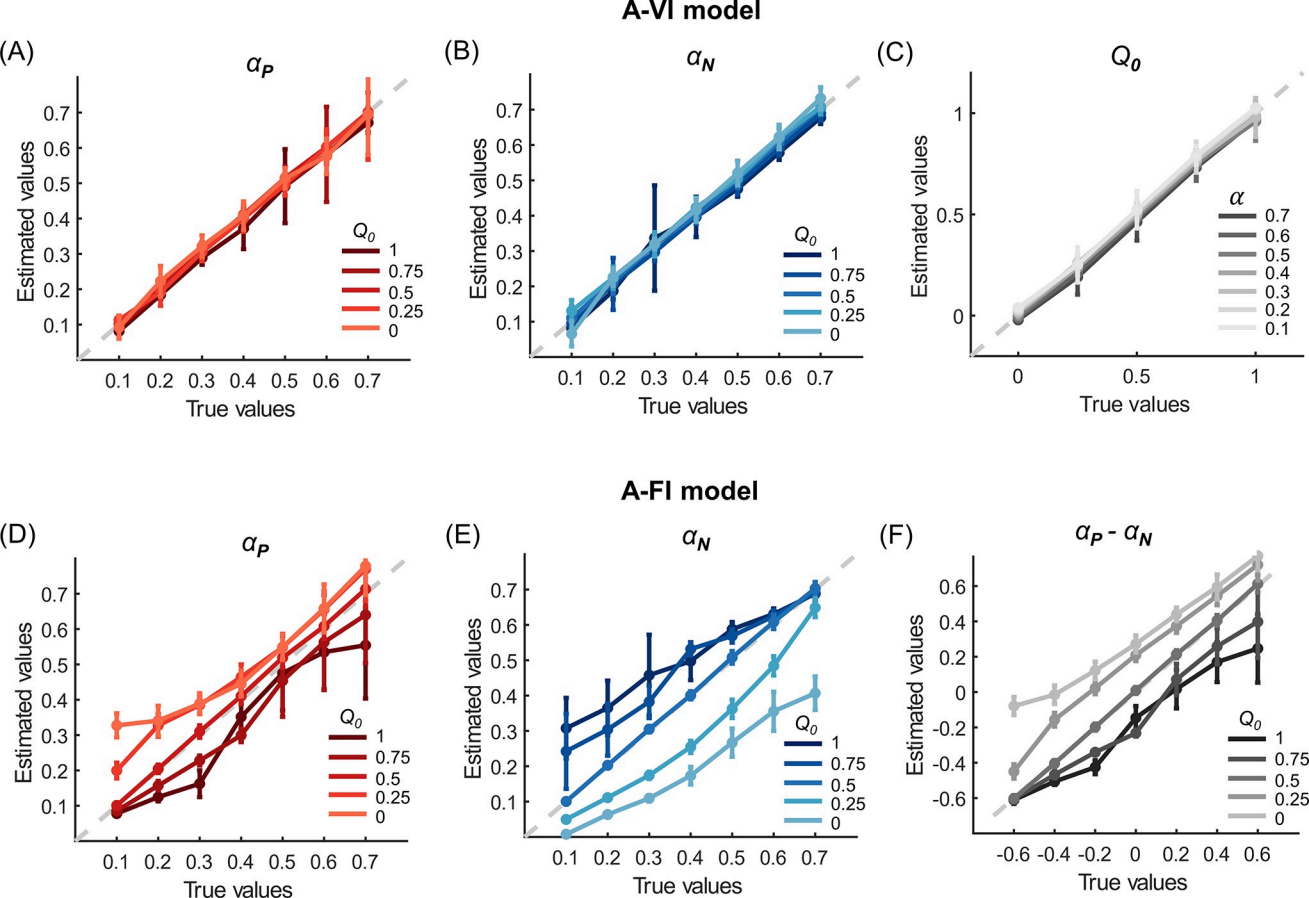
Simulation and parameter recovery for the Gain condition of Experiment 1. Choice data were simulated using different combinations of positive/negative learning rates and initial expectations. The simulated data were then fitted by the A-VI (A-C) and A-FI (D-F) models. The A-VI model faithfully retrieved the underlying parameters (A-C) whereas the A-FI model showed consistent deviations in parameter recovery (D-F). In panels (A-B) and (D-F), different colored (gray) lines represent learning rate recoveries for different *Q*_0_ levels. In panel (C), each gray line represents the recovered *Q*_0_ with different levels of the learning rate (*α*) by grouping *α*_*P*_ and *α*_*N*_ of the same level together. Error bars denote standard deviations across simulated subjects.

As expected, the parameters were well-recovered by the A-VI model for all the parameter combinations (Fig 3A–3C, Gain condition). On the contrary, when fitting without considering the initial expectation differences across subjects (A-FI, *Q*_0_ = 0.5), both the positive and negative learning rates showed a systematic deviation from their true underlying values (Fig 3D and 3E, Gain condition). More specifically, when *Q*_0_<0.5, the positive learning rates were overestimated and the negative learning rates underestimated; whereas the positive learning rates were underestimated and the negative learning rates overestimated when *Q*_0_>0.5. The reason for such biases is due to the fact that when the true *Q*_0_ deviates from the assumed *Q*_0_(0.5), prediction errors caused by the misspecification of initial expectations can only be absorbed by rescaling the learning rates. Further learning rate asymmetry analysis demonstrated this pattern: the learning rate asymmetry (*α*_*P*_−*α*_*N*_) was overestimated when the true initial expectation *Q*_0_<0.5 and underestimated when *Q*_0_>0.5 (Fig 3F, Gain condition). Similar results were also found in the Loss condition (S3 Fig).

**Fig 4:**
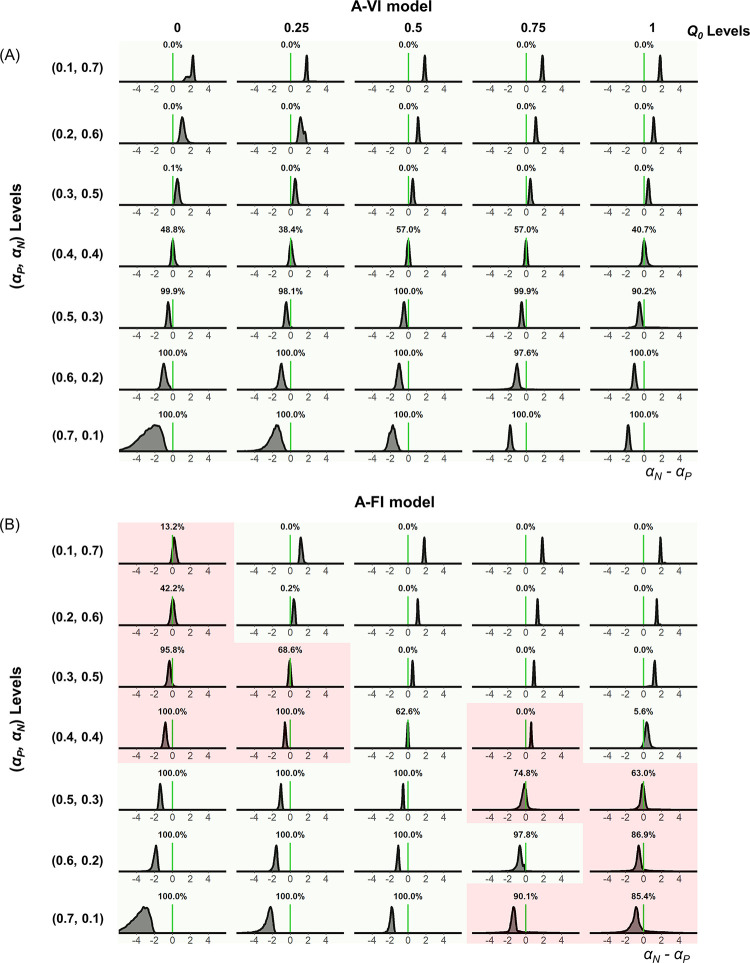
Posterior distribution of learning asymmetry for the Gain condition of Experiment 1. The posterior distribution of *μ*_*δ*_, the hyper mean of the negative learning asymmetry for the A-VI model (A) and A-FI model (B). Light green in each distribution indicates the correct identification of learning asymmetry, whereas red shows the miscategorization for both models (A-VI and A-FI).

We also directly examined the estimated learning asymmetries with the posterior distribution of *μ*_*δ*_, the hyperparameter of the learning asymmetry in the A-VI and A-FI models for the simulated data (Fig 1B). For each combination of the underlying parameters, the estimated *μ*_*δ*_ from the 30 datasets were pooled together to form the posterior distribution of *μ*_*δ*_ (Fig 4, Gain condition). For the A-VI model, the learning asymmetry was correctly recovered for all levels of *Q*_0_ and learning rate pairs (Fig 4A, Gain condition). However, the learning asymmetry was only partially recovered for the A-FI model (Fig 4B, Gain condition). Consistent with the learning rate estimation bias mentioned above, when *Q*_0_<0.5, the estimated positive learning rate tended to be larger than the negative learning rate (even if the true positive and negative learning rates were identical, or the true positive learning rate was smaller than the negative one) (Fig 4B red shaded areas). Likewise, if *Q*_0_>0.5, the estimated negative learning rate tended to be larger than the positive one (Fig 4B red shaded areas). Results of similar patterns were obtained in the Loss condition (S4 Fig).

### Generalization of the initial expectation effect to non-stable learning environment

**Fig 5:**
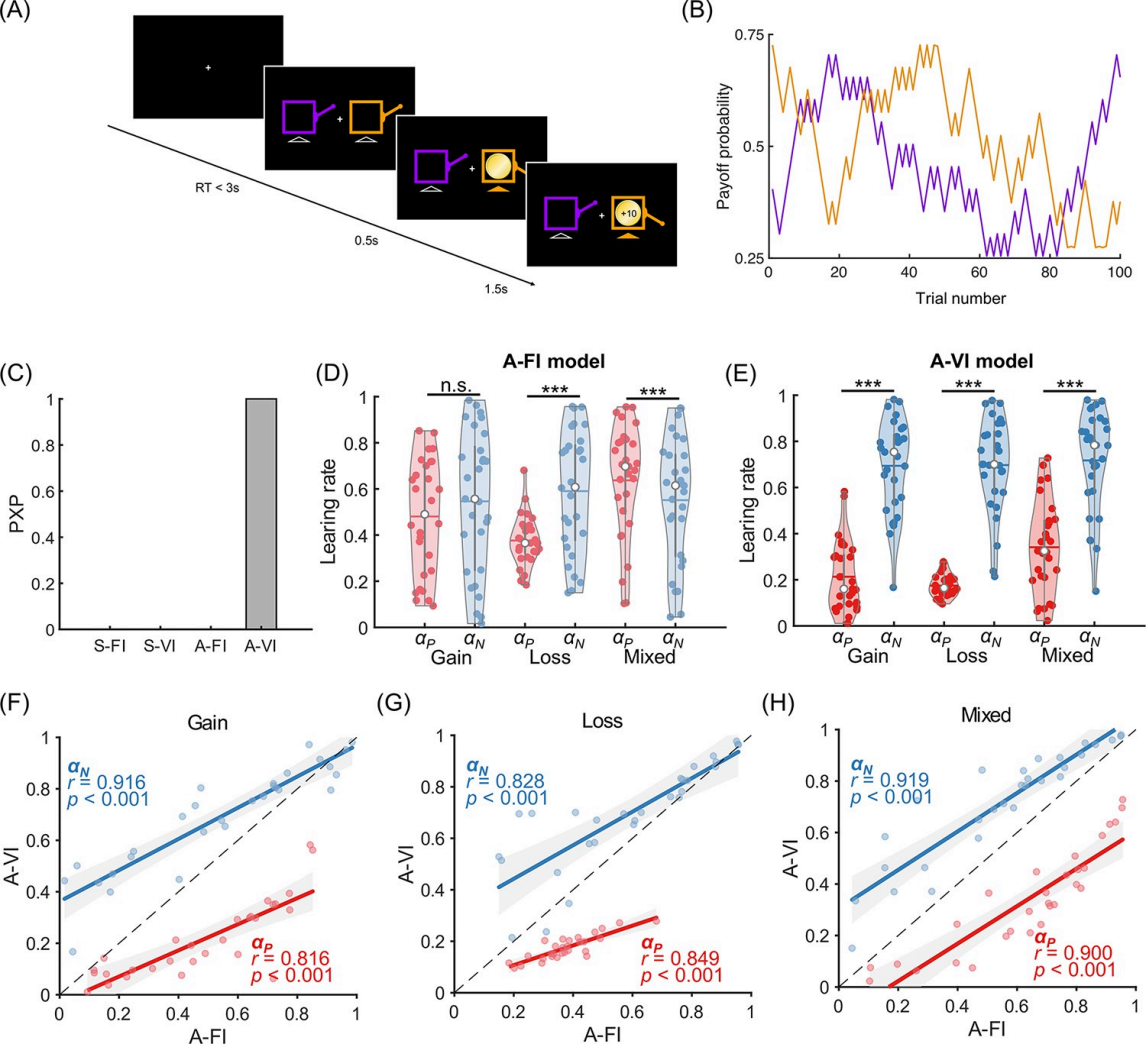
Experiment design and modeling results of Experiment 2. (A) A sample trial for Experiment 2. Participants were asked to choose between two slot machines to maximize their payoffs. (B) Example payoff probability sequences for the two slot machines (purple and orange) slowly evolved across trials. (C) Model comparison results showed that the A-VI model outperformed other candidate models. (D-E) Consistent pattern of learning asymmetry was observed under the A-VI model for the Gain, Loss, and Mixed conditions (E) but not for the A-FI (D) model. (F-H) Both learning rates are positively correlated between A-FI and A-VI models for the Gain (F), Loss (G), and Mixed conditions (H). Asterisks (***) indicate *p* < 0.001 (paired t-test) and n.s. denotes no statistical significance (*p* > 0.05).

**Fig 6:**
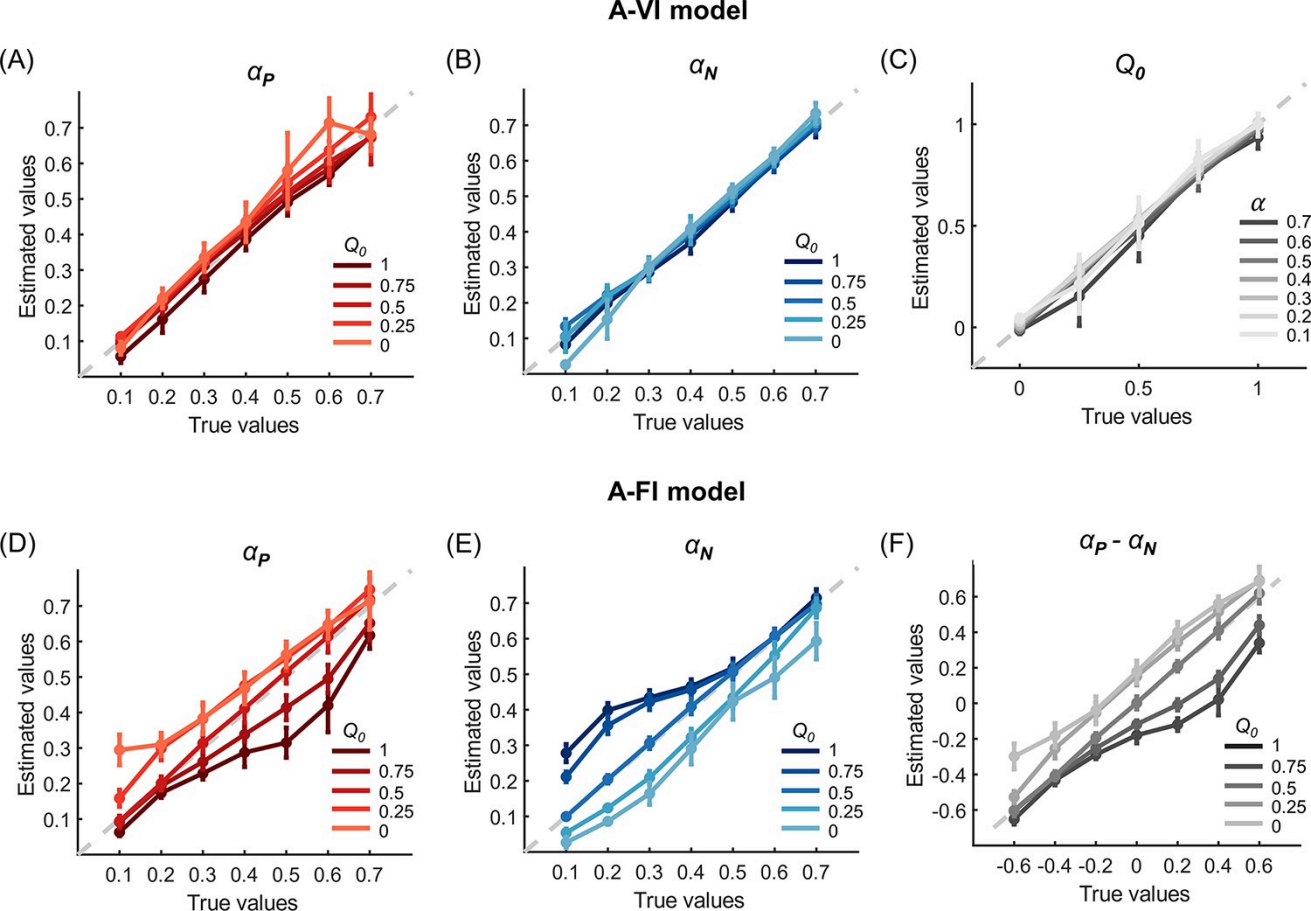
Simulation and parameter recovery for the Gain condition of Experiment 2. Choice data were simulated using different combinations of positive/negative learning rates and initial expectations. The simulated data were then fitted with the A-VI (A-C) and A-FI (D-F) models. The A-VI model faithfully retrieved the underlying parameters (A-C) whereas the A-FI model showed consistent deviations in parameter recovery (D-F). In panels (A-B) and (D-F), different colored (gray) lines represent learning rate recoveries for different *Q*_0_ levels. In panel (C), each gray line represents the recovered *Q*_0_ with different levels of the learning rate (*α*) by grouping *α*_*P*_ and *α*_*N*_ of the same level together. Error bars denote standard deviations across simulated subjects.

**Fig 7:**
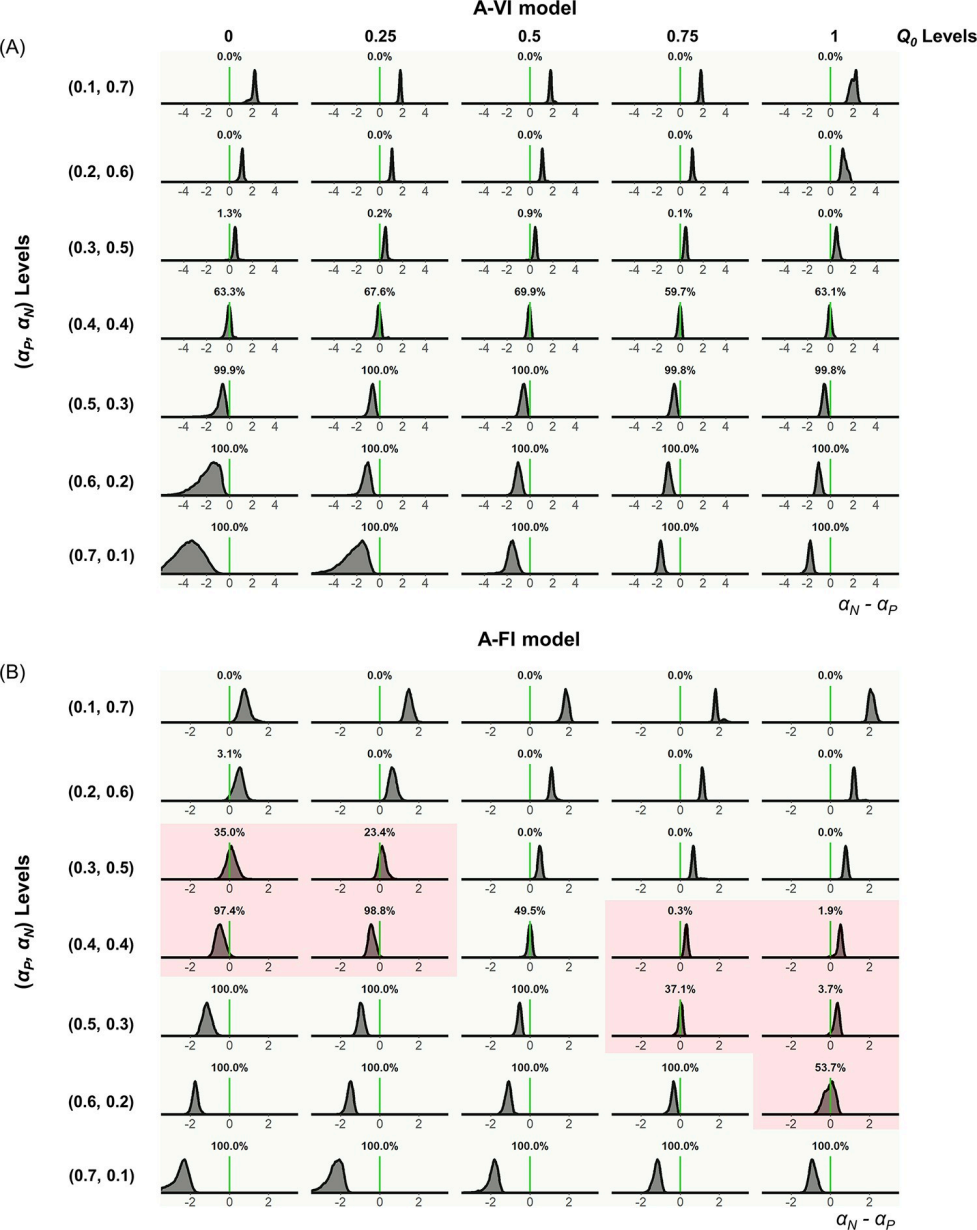
Posterior distribution of learning asymmetry for the Gain condition of Experiment 2. The posterior distribution of *μ*_*δ*_, the hyper mean of the negative learning asymmetry for the A-VI model (A) and A-FI model (B). Light green in each distribution indicates the correct identification of learning asymmetry, whereas red shows the miscategorization for both models (A-VI and A-FI).

To test the obstinate effect of initial expectations on choice behavior, we further collected participants’ choices in a non-stable learning environment (Experiment 2), where the option reinforcement (reward or punishment) probabilities gradually evolved across trials (random walk with boundaries, see Methods) and the learning sequence was longer than that of the stable environment (Fig 5A and 5B). In this experiment, we also included another condition of mixed valence options, where the outcome of an option was either positive (+10 points) or negative (-10 points). 30 subjects participated in this experiment. Similar model fitting procedure was applied, and the model comparison analysis found that the A-VI model outperformed the other three alternatives, with its protected exceedance probability larger than 99.9% (Fig 5C). Again, A-FI and A-VI models produced different learning rate asymmetry (Fig 5D and 5E). While the A-FI model estimation only revealed significant learning asymmetry between positive and negative learning rates in the Loss and Mixed conditions (*p-values* < 0.001, paired t-test) but not in the Gain condition (*p* = 0.161; Fig 5D), the A-VI model showed consistent biased learning pattern across all three conditions, with the negative learning rate significantly larger than the positive learning rate (all *p-value*s < 0.001; Fig 5E). The estimated learning rates from these two models were also significantly correlated in all three conditions (Fig 5F–5H; Gain *α*_*P*_: *r* = 0.816, *p* < 0.001; Gain *α*_*N*_: *r* = 0.916, *p* < 0.001; Loss *α*_*P*_: *r* = 0.849, *p* < 0.001; Loss *α*_*N*_: *r* = 0.828, *p* < 0.001; Mixed *α*_*P*_: *r* = 0.900, *p* < 0.001; Mixed *α*_*N*_: *r* = 0.919, *p* < 0.001). Interestingly, similar to what we found in Experiment 1, the initial values (*Q*_0_) estimated by the A-VI model were also clustered near the lower end of the Q-value range (mean±s.d.: 0.009±0.002 for the Gain, −0.712±0.282 for the Loss, and −0.912±0.064 for the Mixed condition). Similarly, we also ran model simulation and parameter recovery analyses in Experiment 2 (Figs 6 and 7), and the results confirmed that not specifying the initial expectation caused biased estimations of both the positive and negative learning rates: *α*_*P*_ was overestimated and underestimated when *Q*_0_ was smaller or bigger than 0.5, respectively (Fig 6D). *α*_*N*_, however, was underestimated when *Q*_0_<0.5 and overestimated when *Q*_0_>0.5 (Fig 6E). The difference between *α*_*P*_ and *α*_*N*_ was mainly overestimated when *Q*_0_<0.5 and underestimated when *Q*_0_>0.5 (Fig 6F). Finally, the posterior distribution of *μ*_*δ*_ in Experiment 2 confirmed that the learning asymmetry could be correctly identified at different *Q*_0_ levels when *Q*_0_ was treated as an individual parameter (Fig 7A), whereas the mis-specification of learning asymmetry would occur as a by-product of ignoring the heterogeneity of initial expectations (Fig 7B). Again, similar simulation results were found in the Loss and Mixed conditions (S5–S8 Figs).

## Discussion

In both experiments, we tested and verified the hypothesis that the initial expectation has a profound impact on participants’ choice behavior, as opposed to the general assumption that with enough learning trials, the effect of initial expectation would be “washed out”. Interestingly, as a consequence, we also found that learning asymmetry (positive and negative learning rates) estimation can be consistently biased depending on the distance between the assumed and the true underlying initial expectations. We systematically tested these results in both stable (Experiment 1) and slowly evolving random-walk (Experiment 2) probabilistic RL environments. For both experiments, the model with asymmetric learning rates and initial expectation parameters (A-VI) fitted subjects’ behavior best, suggesting the initial expectation parameter could capture additional variance of subjects’ behavior, above and beyond what can be explained by asymmetric learning. Similarly, by adopting the same modeling approach, the re-analyses of data from previous studies [4,28] also revealed the superior performance of our model (A-VI, S9 Fig), suggesting such a learning asymmetry may generalize to other learning environments.

Previous literature has linked state or action values to psychological mechanisms such as incentive salience, which maps “liked” objects or actions to “wanted” ones [32]. This line of research emphasized the critical role played by dopamine in assigning incentive salience to states or actions [38,39]. Other research suggests that such value expectations also affect the strength or vigor of responding in free-operant behaviors [31], possibly with the evolvement of tonic dopamine. The motivational characteristic of action value suggests it is critical not only for generating PE but also for influencing how PE is obtained through choice selection. For example, when subjects were endowed with low expectations to start the gain task and received reward, the rather large positive PE would drive up the action value of the selected option. Therefore, subjects were more likely to stick with the option and missed the opportunity to explore other options. This is indeed what we observed in the equal probability conditions in Experiment 1 (Fig 2E–2H): when subjects’ initial expectations (*Q*_0_) substantially deviated from true action values, negative correlations between *Q*_0_ and the preferred response rates were observed (Fig 2F and 2G); however, such correlation disappeared when the individual *Q*_0_ more aligned with the true action value (Fig 2E and 2H).

It is worth pointing out that the estimated initial action values (*Q*_0_) were lower than the expected returns of the options from the task in all conditions (Gain, Loss, and Mixed) of both experiments. Such a result might indicate attenuated response vigor or a pessimistic judgment bias, both of which have been shown to be related to acute stress, anxiety, and depression symptoms [40,41]. In fact, an augmented Q-value function can take the response vigor (*v*) into account and treat the average reward rate ($\bar{R}$) as an opportunity cost. The new Q-value, therefore, is defined against the ratio of the average rate and response vigor ($\bar{R}/v$) and can be small when the response vigor is low [31]. Although it is beyond the scope of the current study, future research is needed to test how the vigor, acute stress, anxiety, and depression levels are related to the different components (*Q*_0_ and learning rates) of RL [42,43].

It is interesting to note that after removing the shadowing effects of the initial expectation, results from both experiments reveal a consistent negativity bias in learning: people learn faster from negative PEs than from positive ones. This result holds across valence (gain, loss, and mixed) and option reinforcement probability structures (stable and random-walk). Despite recent interests in learning asymmetries in belief, value, and group impression updating [4,5,16,24, 44], questions still remain regarding the direction and magnitude of the asymmetry. Although evidence starts to emerge to support a positivity bias (*α*_*P*_>*α*_*N*_) ranging from the high-level belief update to more elementary forms of updates such as RL [4,5,24], other studies seem to support a negativity bias (*α*_*P*_<*α*_*N*_) in learning [6, 7, 22, 45–47]. One possibility to reconcile such a discrepancy is by considering participants’ beliefs about the causal structure of the environment. For example, it has been shown that if participants infer that the experienced good (or bad) outcomes are due to a hidden cause, rather than the innate uncertainty of the outcome distribution, they would learn relatively less from these outcomes, thus generating the putative negativity (or positivity) bias [16]. Here we propose another possibility: learning asymmetry estimation may be overshadowed by the participants’ initial expectations. Indeed, computational modeling analysis may yield learning asymmetry of different directions depending on the specification of default *Q*_0_, even when learning is in fact symmetric (Figs 3F and 6F).

It should also be noted that the relative ranks of learning rates (positive or negative) across subjects are well preserved, before and after the consideration of initial expectations. In fact, correlation analyses of both the *α*_*P*_ and *α*_*N*_ from the A-FI and A-VI models show they are positively correlated across different conditions in both experiments (Figs 2C, 2D and 5F–5H). However, when inferences are to be drawn about the learning asymmetry, that is, the comparison of *α*_*P*_ and *α*_*N*_, the effect of initial expectations starts to emerge. Previous literature has shown that other factors such as the response autocorrelation might also influence whether learning asymmetry can be identified and proposed model-free methods to mitigate estimation biases [48,49]. Our current study adds to this line of research by demonstrating the necessity of including initial expectations to better characterize subjects’ learning behavior in different learning environments (stable and slowly evolving reinforcement probability), different outcome valences (gain, loss, or mixed conditions) and different lengths of learning sequences (short or long).

In summary, here we demonstrate that initial expectations play a significant role in identifying learning asymmetry in a variety of learning environments, with support from computational modeling, model simulation, and parameter recovery analyses. Our findings help pave the way for future studies about learning asymmetry, which has been implicated in a range of learning and decision making biases in both healthy people [15, 50–52], as well as those who suffer from psychiatric and neurological diseases [53,54].

## Methods

### Ethics statement

The experiments had been approved by the Institutional Review Board of School of Psychological and Cognitive Sciences at Peking University. Formal informed verbal consent was obtained from all the subjects prior to the experiments.

### Subjects

The study consisted of two experiments. 28 subjects participated in Experiment 1 (14 female; mean age 22.3 ± 3.2), of which one participant (male) was excluded from the analysis due to technical problems. 30 subjects participated in Experiment 2 (16 female; mean age 22.1 ± 2.4) and one participant (male) was excluded due to the exclusive selection of the same-side option on the computer screen during the experiment (97%).

### Behavioral tasks

In each experiment, subjects performed a probabilistic instrumental learning task in which they chose between different pairs of visual cues to earn monetary rewards or avoid monetary losses. In Experiment 1, characters from the Agathodaemon alphabet were used as option cues and their associative outcome probabilities were stationary. Outcome valence was manipulated in two conditions: in the Gain condition, the possible outcomes for each cue were either gaining 10 points or zero, whereas, in the Loss condition, outcomes were either losing 10 points or zero. In each condition there were four probability pairs of 40/60%, 25/75%, 25/25%, and 75/75%, respectively. Probability pairs were grouped into mini-blocks, with 32 trials for each mini-block. There is a minimum of 5 seconds rest time between the mini-blocks and a minimum of 20 seconds rest time between conditions. The visual cues and the sequence of probability pairs for each mini-block were randomly assigned across subjects. Participants started with two practice mini-blocks (5 trials each) before the experiment using different visual cues and outcome probabilities. At the end of the experiment, points earned by the participants were converted to monetary payoff using a fixed ratio and participants earned ¥45 on average.

Within each mini-block, a trial started with a fixation cross at the center of the computer screen (1 s), followed by the presentation of the visual cues for the options (maximum 3 s), during which subjects were required to choose either the left or the right option by pressing the corresponding buttons on the keyboard. An arrow appeared under the option for 0.5 s (Fig 1A) to indicate the chosen option immediately after subjects made their choices, followed by the presentation of the selected option outcome of that trial. If subjects responded faster than the 3 s time limit, the remaining time was added to the duration of fixation presentation of the next trial. If no choice was made within the 3 s response time window, a text message “Please respond faster” was displayed for 1.5 s, and subjects needed to complete the trial again.

The task design of Experiment 2 was similar to that of Experiment 1, and subjects were required to choose between two slot machines. The major distinction of Experiment 2 (from Experiment 1) was that the option outcome probabilities followed a random-walk scheme instead of remaining stable [29,55]. At the beginning of the task, option outcome probabilities were independently drawn from a uniform distribution with boundaries of [0.25, 0.75]. Following each trial, the probabilities were diffused either up or down, equiprobably and independently, by adding or subtracting 0.05. The updated probabilities were then reflected off the boundaries [0.25, 0.75] to maintain them within the range. We tested three types of outcome valences in the Gain (+10 points or 0), Loss (-10 points or 0), and Mixed conditions (+10 or -10 points). Each condition consisted of choosing from a pair of slot machines for 100 trials. The color of slot machines was randomly selected, and the order of the three conditions was counterbalanced across subjects.

### Computational models

The Q-learning algorithm has been used extensively to model subjects’ trial-by-trial behavior during learning [1,56–58]. It assumes subjects learn by updating the expected value (*Q* value) for each action based on the prediction error (*δ*). In our study, we allowed the learning rates for positive and negative prediction errors to be different. After every trial *t*, the value of the chosen option is updated as follows:

$$Q_{t+1}=\left\{ \begin{array}{c} Q_{t}+\alpha_{P}∙(r_{t}-Q_{t}),if\;\delta_{t}\geq 0\\ Q_{t}+\alpha_{N}∙(r_{t}-Q_{t}),if\;\delta_{t}<0 \end{array}\right.$$ (1)

The term *r*_*t*_−*Q*_*t*_ is the prediction error (*δ*_*t*_) in trial *t* and we set the reward, *r*_*t*_ = -1, 0, and 1 for +10 points, 0, and -10 points, respectively. *α*_*P*_ and *α*_*N*_ are the positive and negative learning rates and are constrained to the range of [0, 1]. The initial expectation for each option, *Q*_0_, is set as a free parameter, constrained to the range between the worst and the best outcome of that option. We assumed that the initial expectations for both options in each mini-block were the same for each individual. We refer to this model as the asymmetric RL model with variable initial expectations (A-VI).

The probability of choosing one option over the other is described by the softmax function, with the inverse temperature *β* constrained within [0, 20]:

$$p(c_{t}=1)=\frac{1}{1+e^{-\beta ∙[Q_{t(L)}-Q_{t(R)}]}}$$ (2)

Here, *Q*_*t*(*L*)_ and *Q*_*t*(*R*)_ are the *Q* values for the left and right options in trial *t*. We also considered other variant models of RL. The first one is A-FI, where the initial expectations *Q*_0_ are set as the mean outcomes in the Gain, Loss, and Mixed conditions (0.5, -0.5, and 0) respectively, corresponding to an initial expectation of 50% chance of receiving either outcome (A-FI model with *Q*_0_ = 0 for all the conditions was also tested, see S1 and S2 Tables). The second one is S-VI, where the learning rates for the positive and negative prediction errors are the same (*α*_*P*_ = *α*_*N*_). The last one is S-FI, with identical learning rates for positive and negative prediction errors and *Q*_0_ set as the mean outcomes for each condition (S-FI model with *Q*_0_ = 0 for all the conditions was also tested, see S1 and S2 Tables).

### Bayesian hierarchical modeling procedure and model comparison

We applied a Bayesian hierarchical modeling procedure to fit the models. In contrast to the traditional point estimate method, such as the maximum likelihood approach, the Bayesian hierarchical method generates the posterior distribution of the parameters at both the individual and the group levels in a mutually constraining fashion to provide a more stable and reliable parameter estimation [59–61]. Take the example of the A-VI model (Fig 1B), *r*_*i*,*t*−1_ refers to the outcome received by subject *i* at trial *t*−1 and *c*_*i*,*t*_ is the choice of subject *i* at trial *t*. The individual-level parameters were transformed using the Φ transformation, the cumulative density function of the standard normal distribution, to constrain the parameter values in their corresponding boundaries. In order to directly capture the effect of the learning rate difference [61, 62], we modeled the negative learning rate as the sum of the positive learning rate and the difference between negative and positive learning rates. Specifically, for each parameter *θ* (*θ*∈{*Q*_0_, *α*_*P*_, *β*}) with [*θ*_*min*_, *θ*_*max*_] as its boundary, $\theta =\theta_{min}+\Phi (\theta^{\prime })\times (\theta_{max}-\theta_{min})$. Parameters *θ*′ were drawn from hyper normal distributions with mean *μ*_*θ*′_ and standard deviation *σ*_*θ*′_. A normal prior was assigned to the hyper means *μ*_*θ*′_~*N*(0, 2) and a half-Cauchy prior to the hyper standard deviations *σ*_*θ*′_~*C*(0, 5). The negative learning rate was specified as $\alpha_{N}=\Phi (\alpha_{P}^{\prime }+\delta )$, where *δ* was set the same as *θ*′. The three alternative models were specified in a similar manner. Data from different outcome valence conditions were modeled separately.

Model fitting was performed using R (v4.2.2) and RStan (v2.21.2). For each model, 6000 samples were collected after a burn-in of 4000 samples on each of the four chains, leading to a total of 24,000 samples collected for each parameter (representing the posterior distribution of the corresponding parameter). For each parameter, we computed a trimmed mean by discarding 10% samples from each side to obtain the robust estimation of the corresponding parameter [63].

Given the parameter samples, we computed deviance information criterion (DIC) for each model and used it to compare our candidate models’ performances [13]. We further calculated the protected exceedance probability (PXP), indexing the probability that a specific model is the best among the candidate models, based on the group-level Bayesian model selection method to identify the best model [36,37].

### Model simulations and parameter recovery

To test the robustness of our results, we performed a comprehensive parameter recovery analysis. For each task (stable or random-walk probability scheme), we generated hypothetical choices using the best performing model (A-VI model) with different initial expectation levels and different learning rate levels. We tested the parameter recovery of the Gain (Figs 3 and 4) and Loss (S3 and S4 Figs) conditions for Experiment 1, and the Gain (Figs 6 and 7), Loss (S5 and S6 Figs), and Mixed (S7 and S8 Figs) conditions for Experiment 2, respectively. For example, we considered five levels (0, 0.25, 0.5, 0.75, and 1) of the initial expectation (*Q*_0_) and seven pairs [(0.1, 0.7), (0.2, 0.6), (0.3, 0.5), (0.4, 0.4), (0.5, 0.3), (0.6, 0.2) and (0.7, 0.1)] of positive and negative learning rates (*α*_*P*_, *α*_*N*_) to roughly match the learning rate range we observed for the Gain condition in both experiments (Figs 2A, 2B, 5D and 5E). For each combination of the initial expectation and learning rates, we simulated 30 datasets, leading to a total of 1050 (35 x 30 *Q*_0_ and learning rates combinations) datasets for each task. Each dataset consists of 30 hypothetical subjects. Based on our model fitting results, we set the inverse temperature parameter *β* to 10 for all datasets (Figs 3, 4, 6 and 7), and we found that setting *β* to a lower value of 5 yielded similar results. For each dataset, we fitted models with and without parameterizing the initial expectation (*Q*_0_ was fixed to 0.5, -0.5, and 0 for the Gain, Loss, and Mixed conditions, respectively) using the same Bayesian model fitting method described above.

## Supporting information

S1 Table Model Deviance Information Criterion (DIC). Model fitting results. Models 1, 3, 4 & 6 were reported in the main results. We also considered fixed *Q*_0_ models (S-FI and A-FI) where the *Q*_0_ was fixed to 0 across the Gain, Loss, and Mixed conditions instead of the expected outcome value (models S-FI’ and A-FI’) for each condition. Across two experiments, the A-VI model (M6) consistently performed better than all the other candidates with the PXP > 0.99 for both experiments.
(TIF)

S2 Table Model estimated parameters (mean±s.d.). (TIF)

S1 Fig Participants’ performance in Experiment 1. Participants’ correct choice rates across trials in both the Gain (A) and the Loss (B) conditions. Across two conditions, participants achieved higher correction rates as learning proceeded and their performance was better in the 25–75% block than in the 40–60% block.
(TIF)

S2 Fig The association between learning rates and *Q*_0_ in Experiment 1. Across both the Gain and Loss conditions, no significant correlation was observed between learning rates and *Q*_0_, indicating that learning rates and *Q*_0_ might have independent contributions to the individual differences in learning across participants.
(TIF)

S3 Fig Simulation and parameter recovery for the Loss condition in Experiment 1. Choice data were simulated using different combinations of positive/negative learning rates and initial expectations. The simulated data were then fitted by the A-VI (A-C) and A-FI (D-F) models. The A-VI model faithfully retrieved the underlying parameters (A-C) whereas the A-FI model showed consistent deviations in parameter recovery (D-F). In panels (A-B) and (D-F), different colored (gray) lines represent learning rate recoveries for different *Q*_0_ levels. In panel (C), each gray line represents the recovered *Q*_0_ with different levels of the learning rate (*α*) by grouping *α*_*P*_ and *α*_*N*_ of the same level together. Error bars denote standard deviations across simulated subjects.
(TIF)

S4 Fig Posterior distribution of learning asymmetry for the Loss condition in Experiment 1. The posterior distribution of *μ*_*δ*_, the hyper mean of the negative learning asymmetry for the A-VI model (A) and A-FI model (B). Light green in each distribution indicates the correct identification of learning asymmetry, whereas red shows the miscategorization for both models (A-VI and A-FI).
(TIF)

S5 Fig Simulation and parameter recovery for the Loss condition in Experiment 2. Choice data were simulated using different combinations of positive/negative learning rates and initial expectations. The simulated data were then fitted by the A-VI (A-C) and A-FI (D-F) models. The A-VI model faithfully retrieved the underlying parameters (A-C) whereas the A-FI model showed consistent deviations in parameter recovery (D-F). In panels (A-B) and (D-F), different colored (gray) lines represent learning rate recoveries for different *Q*_0_ levels. In panel (C), each gray line represents the recovered *Q*_0_ with different levels of the learning rate (*α*) by grouping *α*_*P*_ and *α*_*N*_ of the same level together. Error bars denote standard deviations across simulated subjects.
(TIF)

S6 Fig Posterior distribution of learning asymmetry for the Loss condition of Experiment 2. The posterior distribution of *μ*_*δ*_, the hyper mean of the negative learning asymmetry for the A-VI model (A) and A-FI model (B). Light green in each distribution indicates the correct identification of learning asymmetry, whereas red shows the miscategorization for both models (A-VI and A-FI).
(TIF)

S7 Fig Simulation and parameter recovery for the Mixed condition in Experiment 2. Choice data were simulated using different combinations of positive/negative learning rates and initial expectations. The simulated data were then fitted by the A-VI (A-C) and A-FI (D-F) models. The A-VI model faithfully retrieved the underlying parameters (A-C) whereas the A-FI model showed consistent deviations in parameter recovery (D-F). Error bars denote standard deviations across simulated subjects. In panels (A-B) and (D-F), different colored (gray) lines represent learning rate recoveries for different *Q*_0_ levels. In panel (C), each gray line represents the recovered *Q*_0_ with different levels of the learning rate (*α*) by grouping *α*_*P*_ and *α*_*N*_ of the same level together.
(TIF)

S8 Fig Posterior distribution of learning asymmetry for the Mixed condition of Experiment 2. The posterior distribution of *μ*_*δ*_, the hyper mean of the negative learning asymmetry for the A-VI model (A) and A-FI model (B). Light green in each distribution indicates the correct identification of learning asymmetry, whereas red shows the miscategorization for both models (A-VI and A-FI).
(TIF)

S9 Fig A-VI and A-FI modeling results from previous datasets. We selected publications in which the behavioral data were publicly available and the experimental designs were similar to ours and tested the consistency of our model performance [4,26]. The first dataset (N = 20) was from Palminteri et al.,2017 [28], in which four pairs of visual stimuli (24 trials per pair) were assigned with reward probabilities of 0.5/0.5, 0.75/0.25, 0.25/0.75 and 0.83/0.17). The second dataset (N = 50) was from Lefebvre et al.,2017 [4], where similar experimental paradigms were used except that the reward probabilities for the visual stimuli were slightly different (0.25/0.25, 0.75/0.75, 0.25/0.75 and 0.75/0.25, gain trials only). We fitted the behavioral data of the two datasets with two asymmetric learning rate models (A-VI and A-FI). The model fitting and comparison results suggest that the A-VI model performed better than the A-FI model (A and C). Without the initial value term, we found similar positive asymmetry biases as reported before (panels B and D). However, this pattern reversed when the initial value term was included in the A-VI model (interaction effect p-values < 0.001 for panels B and D).
(TIF)
