# Supplementary figures and images for: The shadowing effect of initial expectation on learning asymmetry
Source: PLoS Comput Biol. 2023 Jul 24;19(7):e1010751. doi: 10.1371/journal.pcbi.1010751 (PMC10399892; doi:10.1371/journal.pcbi.1010751)

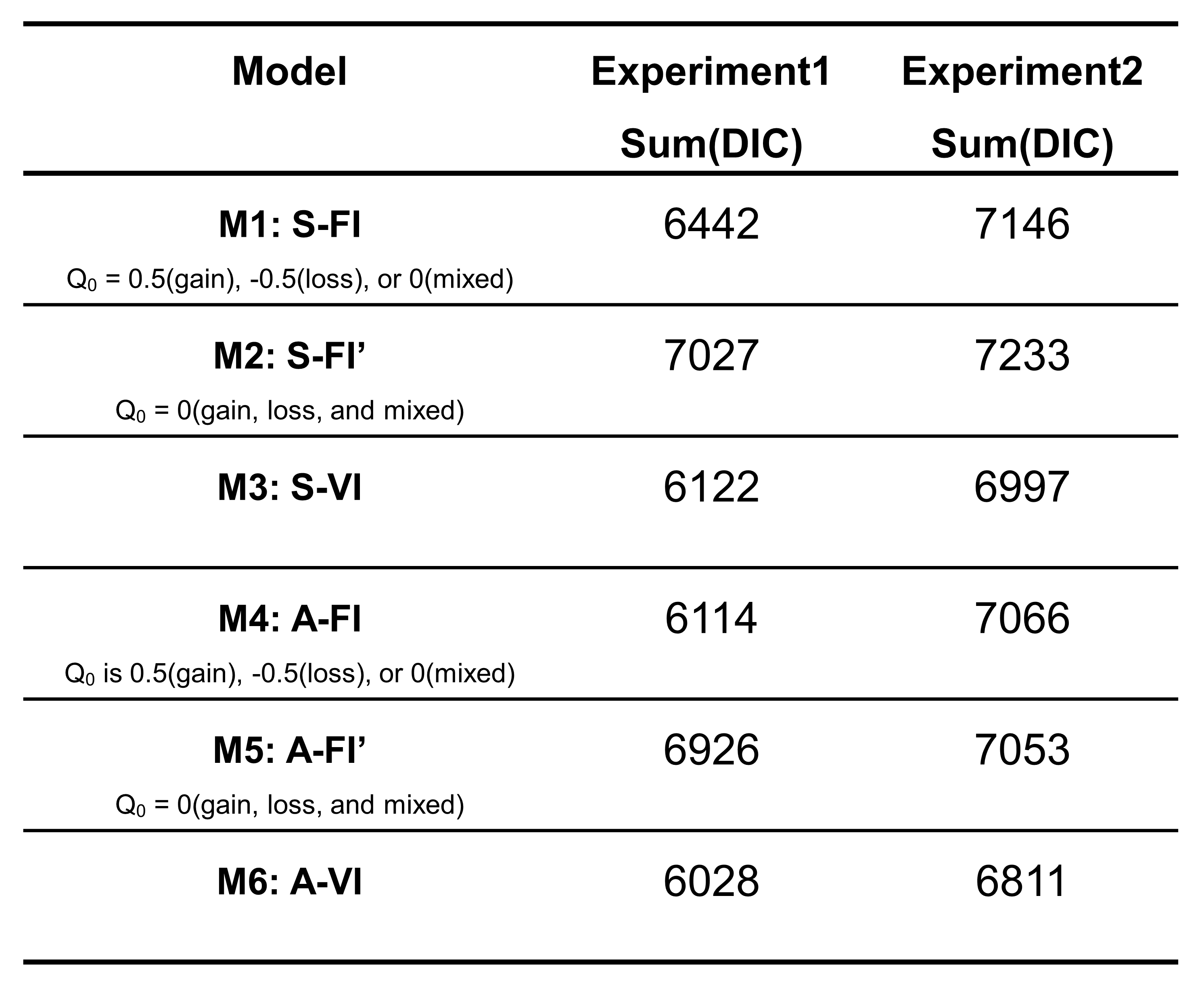

Supplement: S1 Table — Model fitting results. Models 1, 3, 4 & 6 were reported in the main results. We also considered fixed Q0 models (S-FI and A-FI) where the Q0 was fixed to 0 across the Gain, Loss, and Mixed conditions instead of the expected outcome value (models S-FI’ and A-FI’) for each condition. Across two experiments, the A-VI model (M6) consistently performed better than all the other candidates with the PXP > 0.99 for both experiments. (TIF) [file pcbi.1010751.s001.TIF]

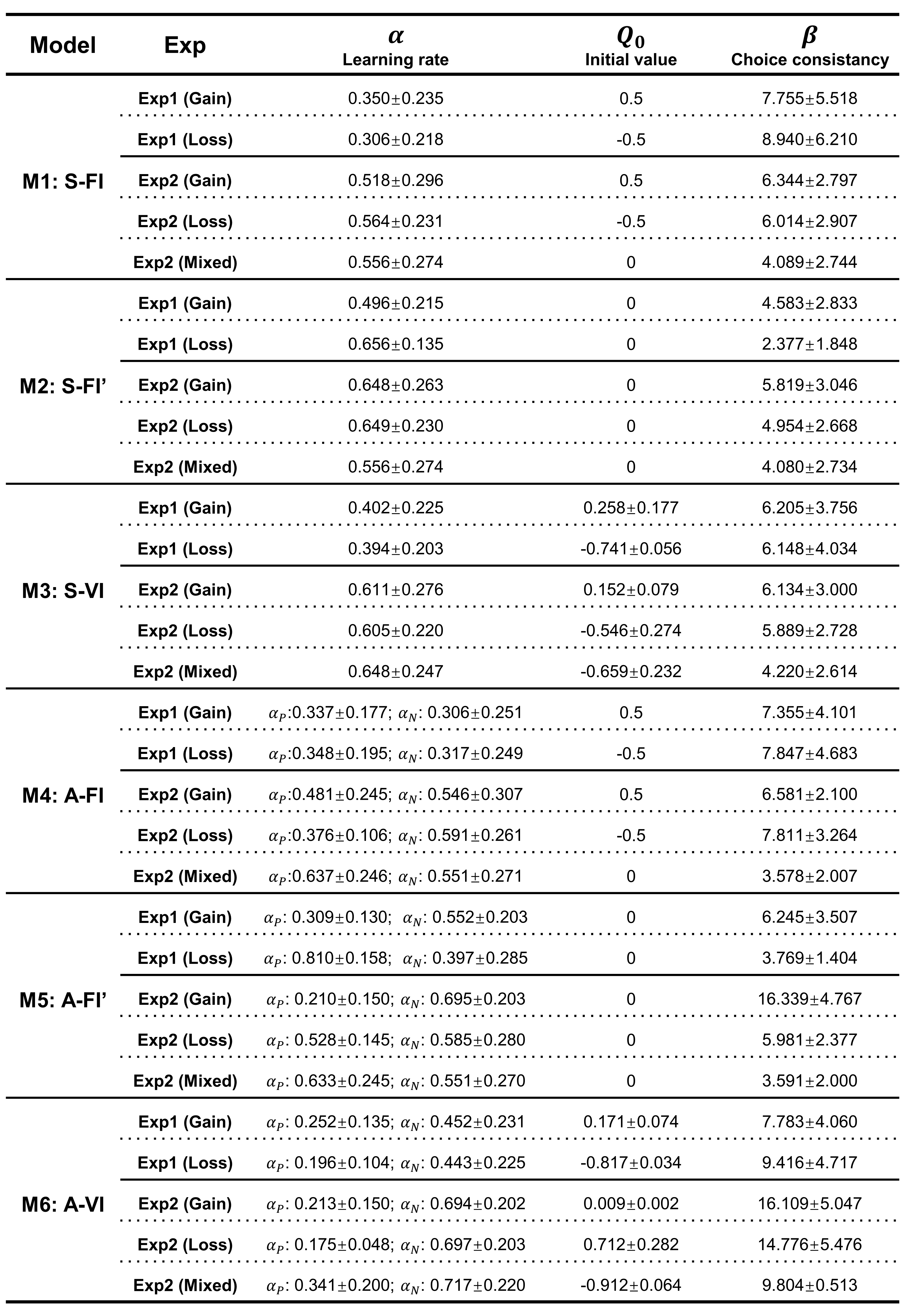

Supplement: S2 Table — (TIF) [file pcbi.1010751.s002.TIF]

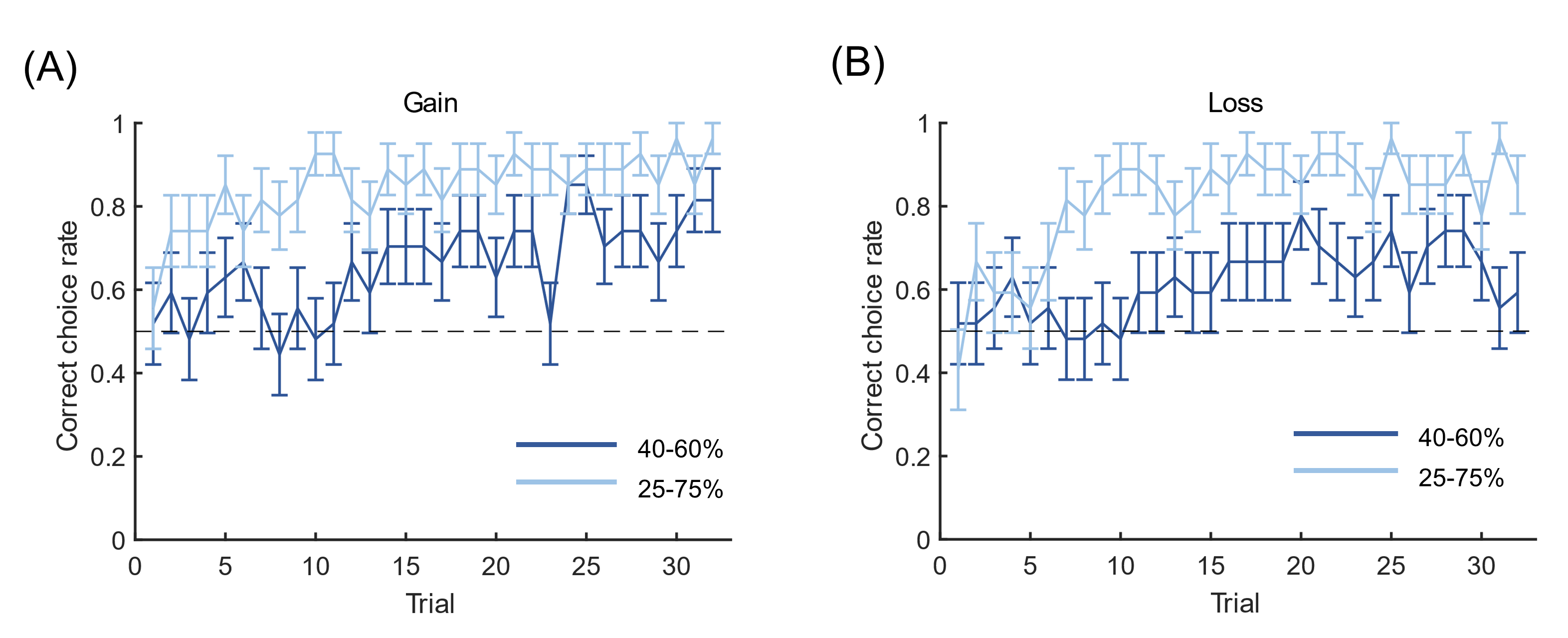

Supplement: S1 Fig — Participants’ correct choice rates across trials in both the Gain (A) and the Loss (B) conditions. Across two conditions, participants achieved higher correction rates as learning proceeded and their performance was better in the 25–75% block than in the 40–60% block. (TIF) [file pcbi.1010751.s003.TIF]

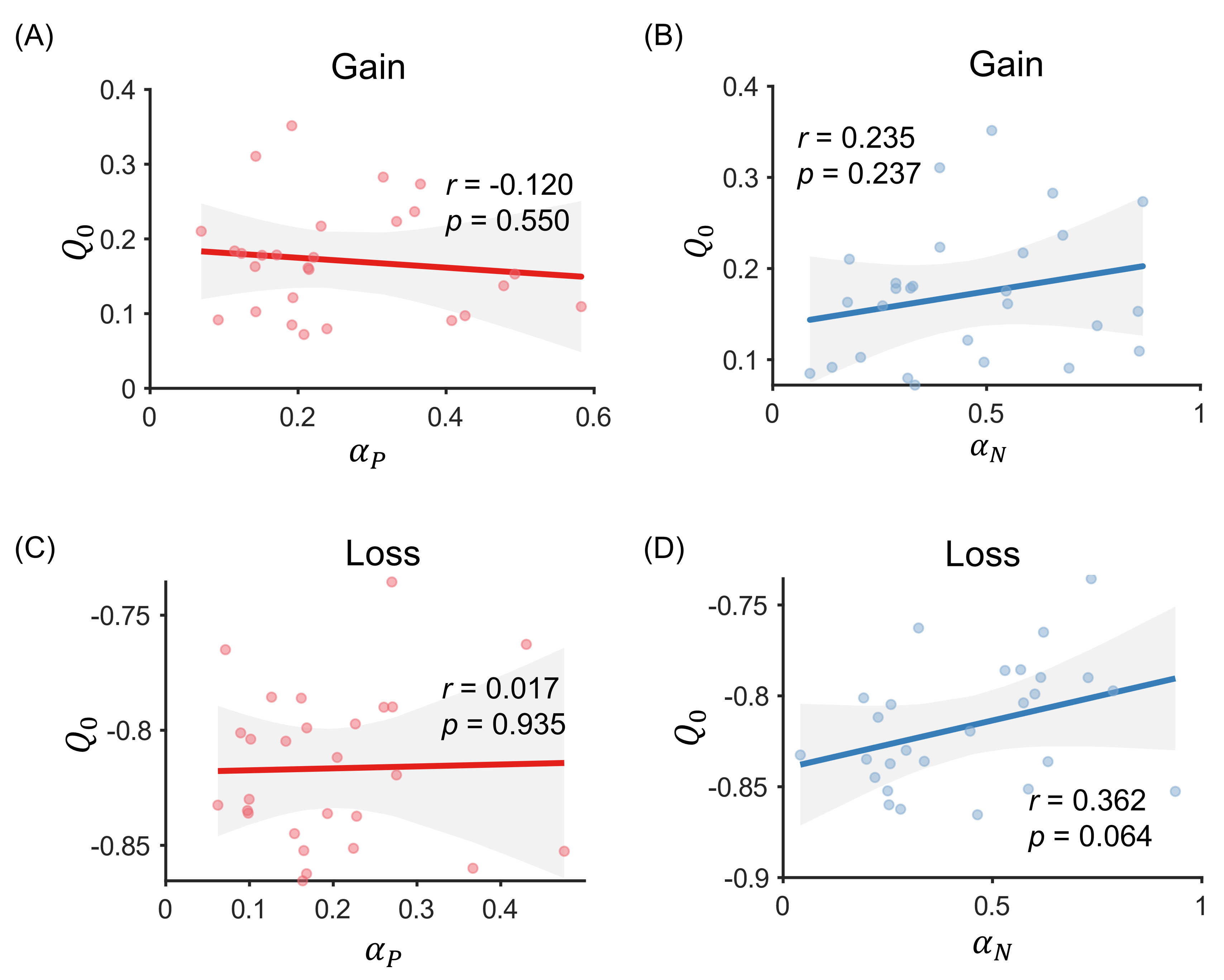

Supplement: S2 Fig — Across both the Gain and Loss conditions, no significant correlation was observed between learning rates and Q0, indicating that learning rates and Q0 might have independent contributions to the individual differences in learning across participants. (TIF) [file pcbi.1010751.s004.TIF]

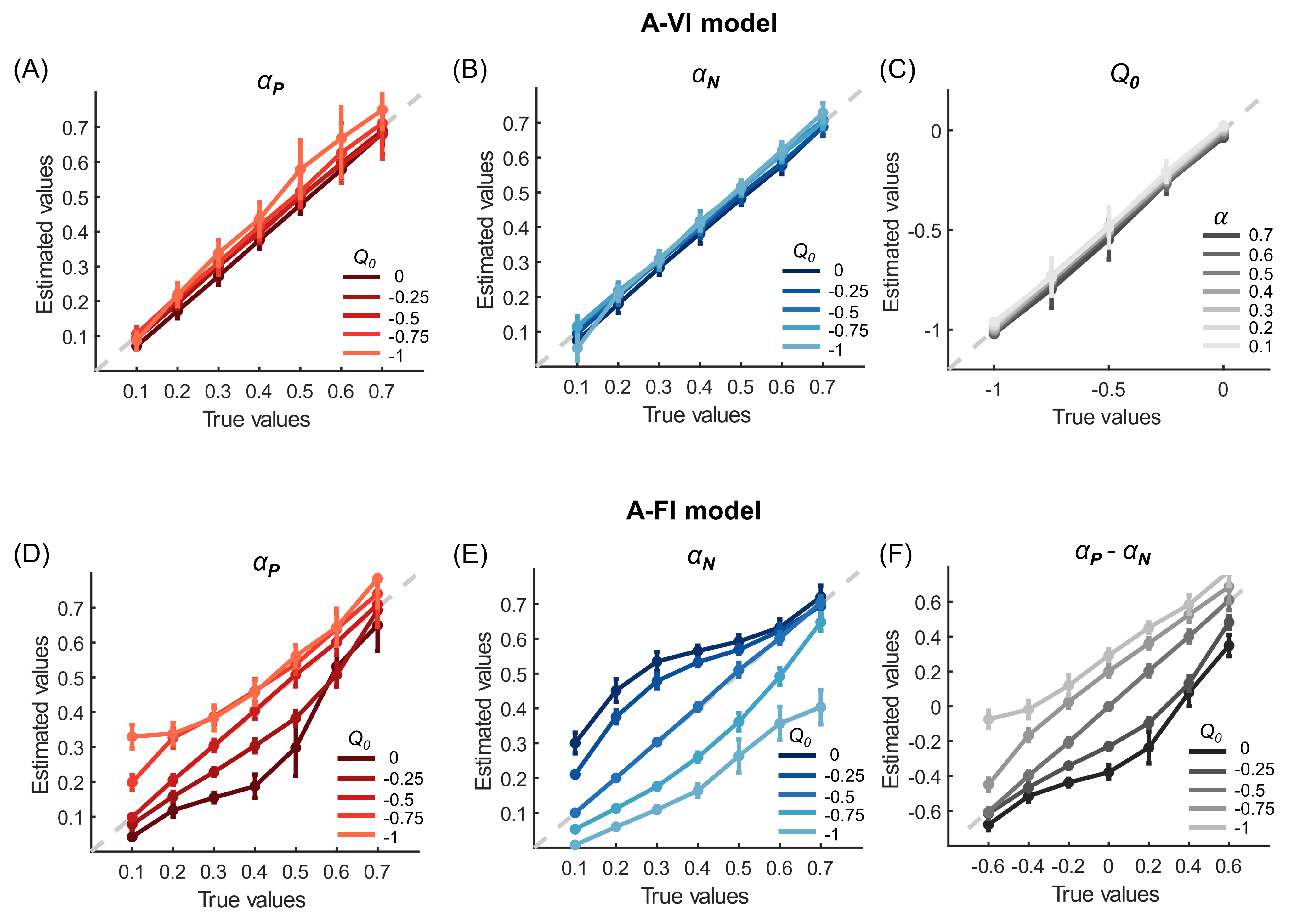

Supplement: S3 Fig — Choice data were simulated using different combinations of positive/negative learning rates and initial expectations. The simulated data were then fitted by the A-VI (A-C) and A-FI (D-F) models. The A-VI model faithfully retrieved the underlying parameters (A-C) whereas the A-FI model showed consistent deviations in parameter recovery (D-F). In panels (A-B) and (D-F), different colored (gray) lines represent learning rate recoveries for different Q0 levels. In panel (C), each gray line represents the recovered Q0 with different levels of the learning rate (α) by grouping αP and αN of the same level together. Error bars denote standard deviations across simulated subjects. (TIF) [file pcbi.1010751.s005.TIF]

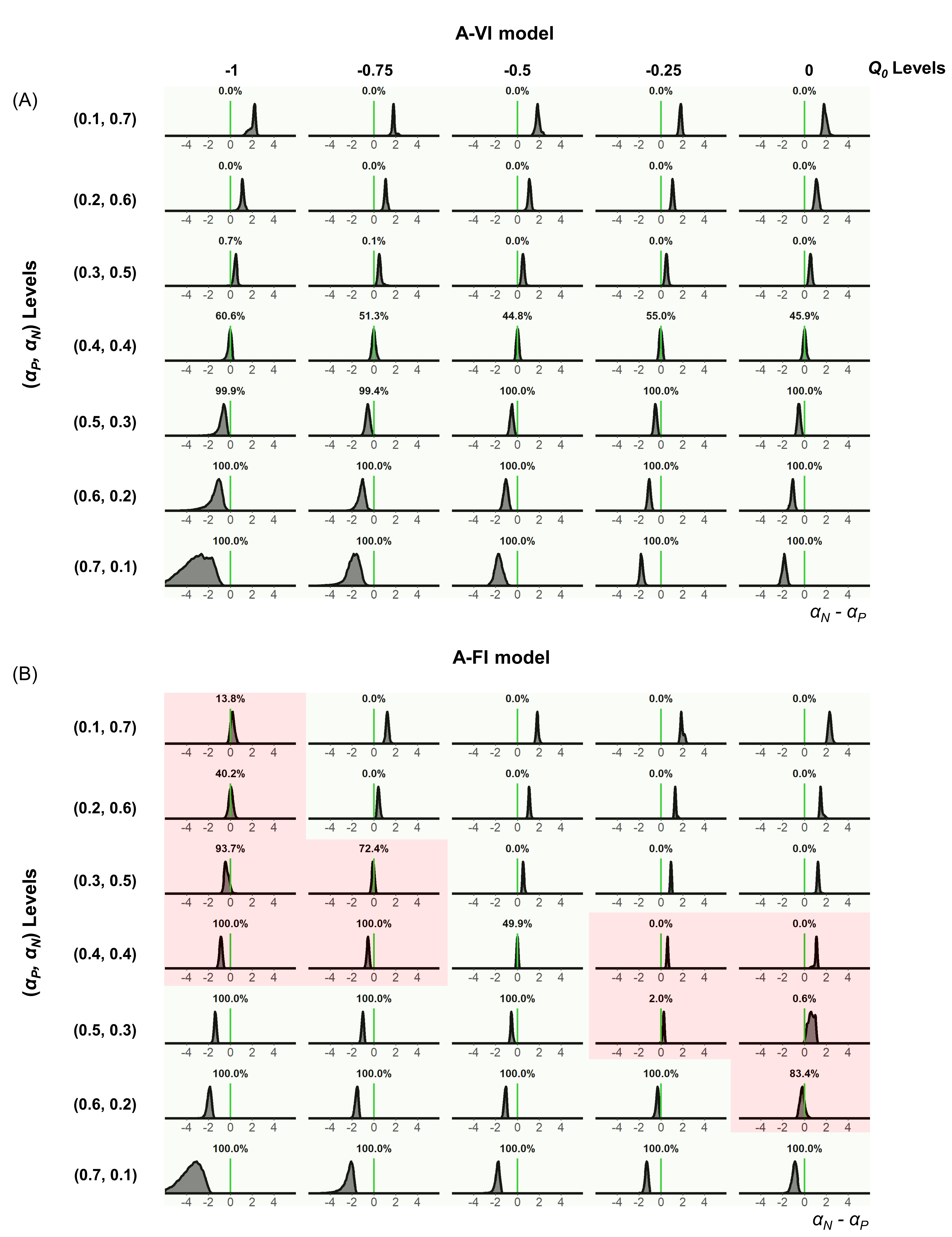

Supplement: S4 Fig — The posterior distribution of μδ, the hyper mean of the negative learning asymmetry for the A-VI model (A) and A-FI model (B). Light green in each distribution indicates the correct identification of learning asymmetry, whereas red shows the miscategorization for both models (A-VI and A-FI). (TIF) [file pcbi.1010751.s006.TIF]

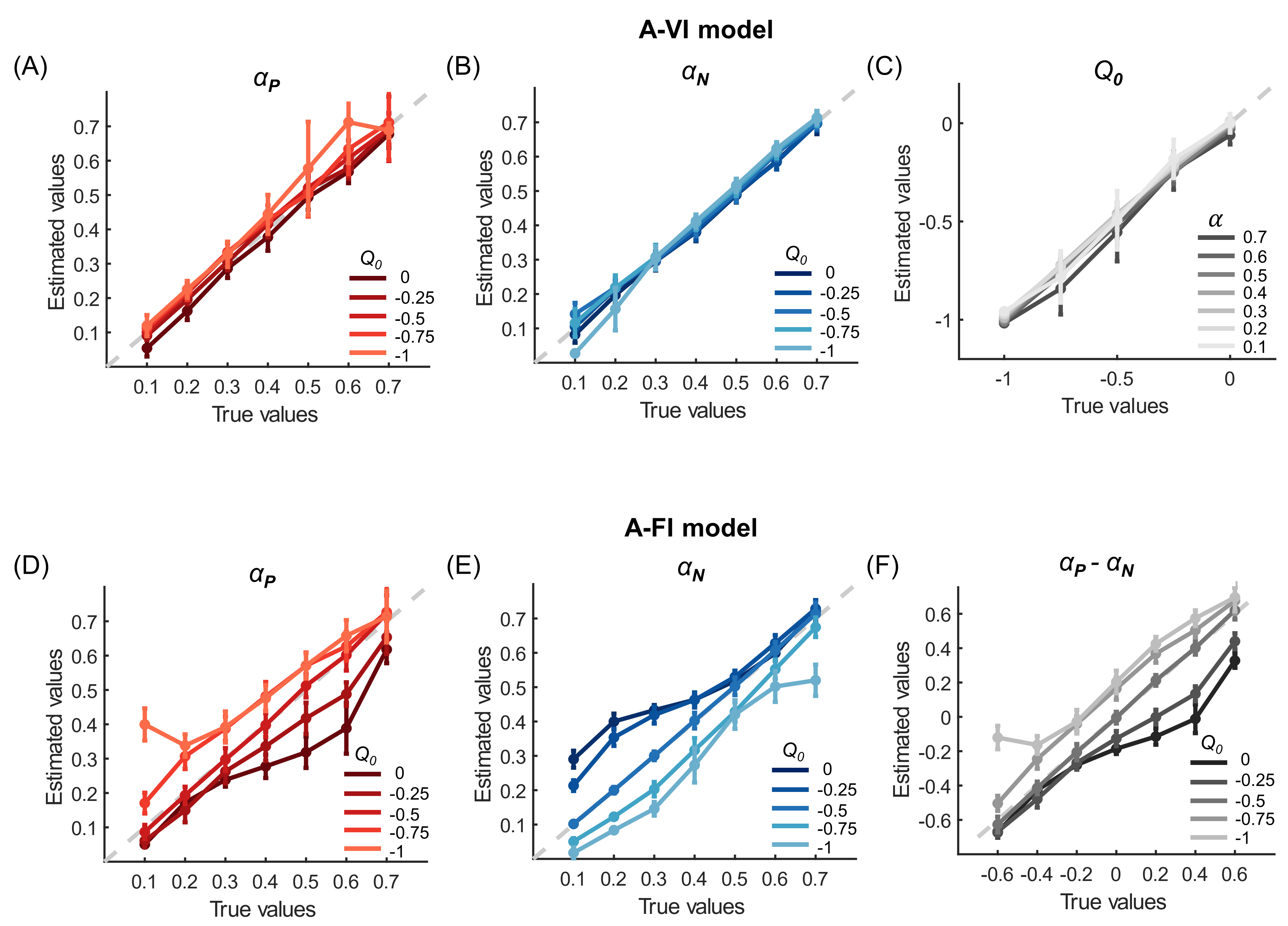

Supplement: S5 Fig — Choice data were simulated using different combinations of positive/negative learning rates and initial expectations. The simulated data were then fitted by the A-VI (A-C) and A-FI (D-F) models. The A-VI model faithfully retrieved the underlying parameters (A-C) whereas the A-FI model showed consistent deviations in parameter recovery (D-F). In panels (A-B) and (D-F), different colored (gray) lines represent learning rate recoveries for different Q0 levels. In panel (C), each gray line represents the recovered Q0 with different levels of the learning rate (α) by grouping αP and αN of the same level together. Error bars denote standard deviations across simulated subjects. (TIF) [file pcbi.1010751.s007.TIF]

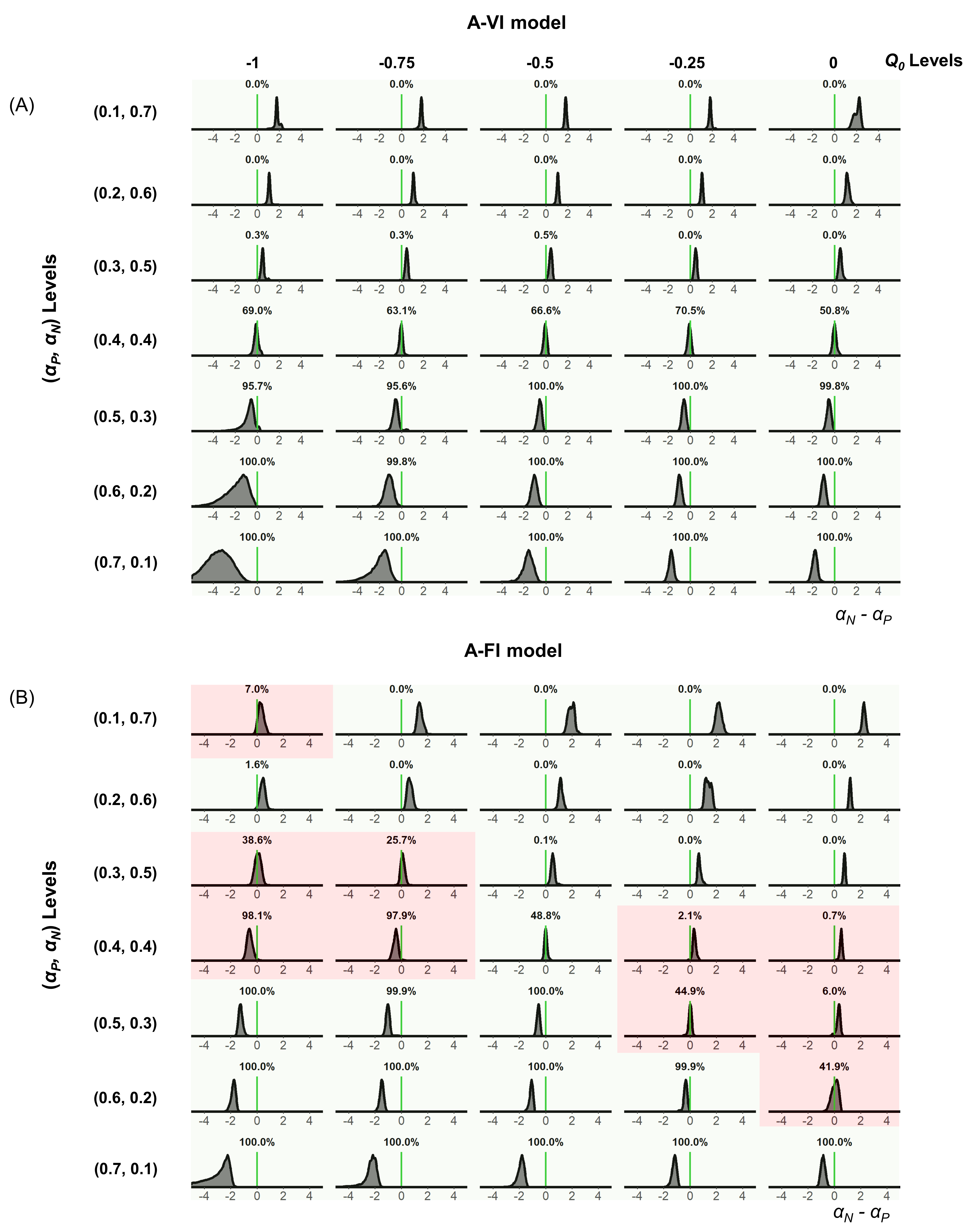

Supplement: S6 Fig — The posterior distribution of μδ, the hyper mean of the negative learning asymmetry for the A-VI model (A) and A-FI model (B). Light green in each distribution indicates the correct identification of learning asymmetry, whereas red shows the miscategorization for both models (A-VI and A-FI). (TIF) [file pcbi.1010751.s008.TIF]

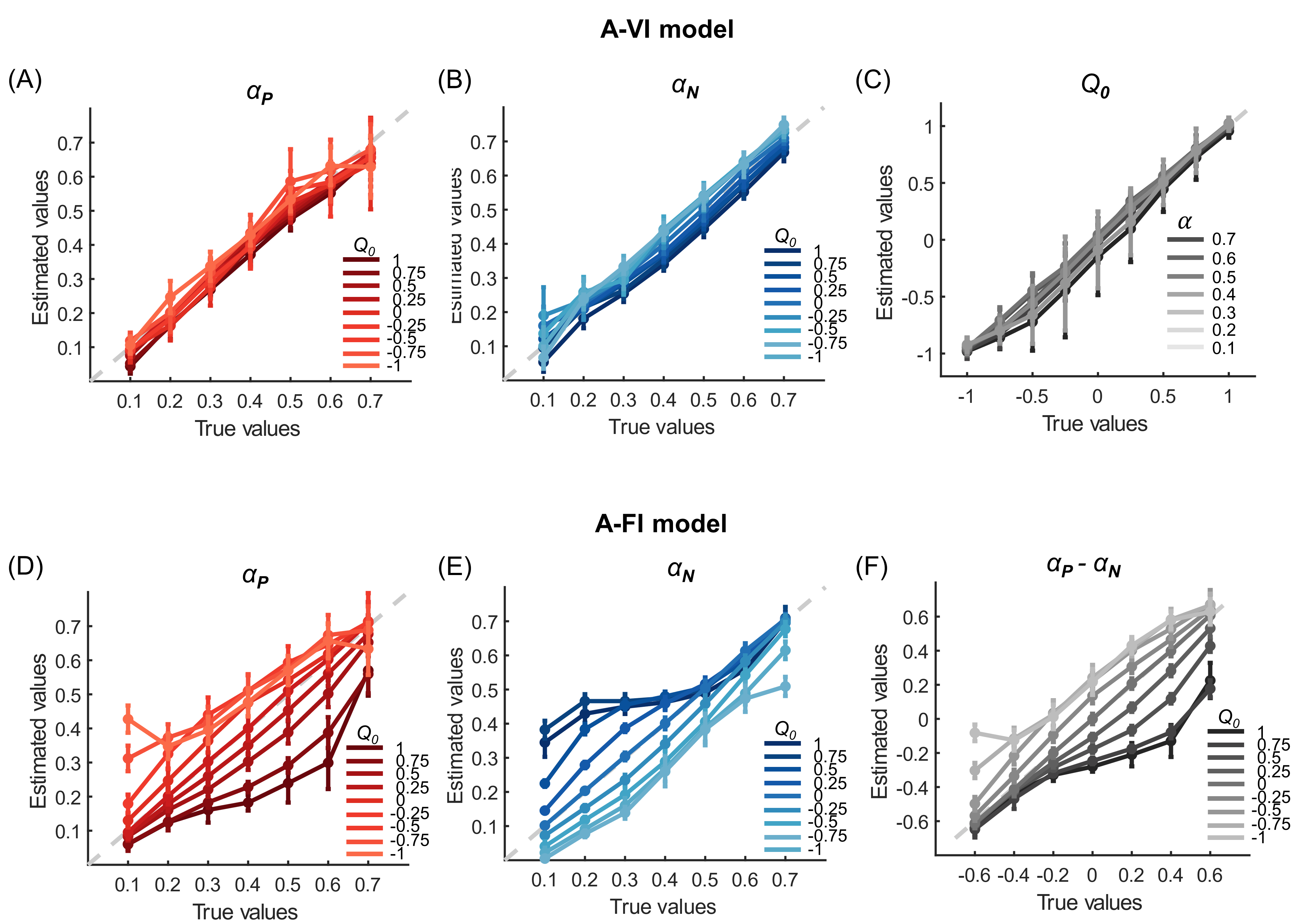

Supplement: S7 Fig — Choice data were simulated using different combinations of positive/negative learning rates and initial expectations. The simulated data were then fitted by the A-VI (A-C) and A-FI (D-F) models. The A-VI model faithfully retrieved the underlying parameters (A-C) whereas the A-FI model showed consistent deviations in parameter recovery (D-F). Error bars denote standard deviations across simulated subjects. In panels (A-B) and (D-F), different colored (gray) lines represent learning rate recoveries for different Q0 levels. In panel (C), each gray line represents the recovered Q0 with different levels of the learning rate (α) by grouping αP and αN of the same level together. (TIF) [file pcbi.1010751.s009.TIF]

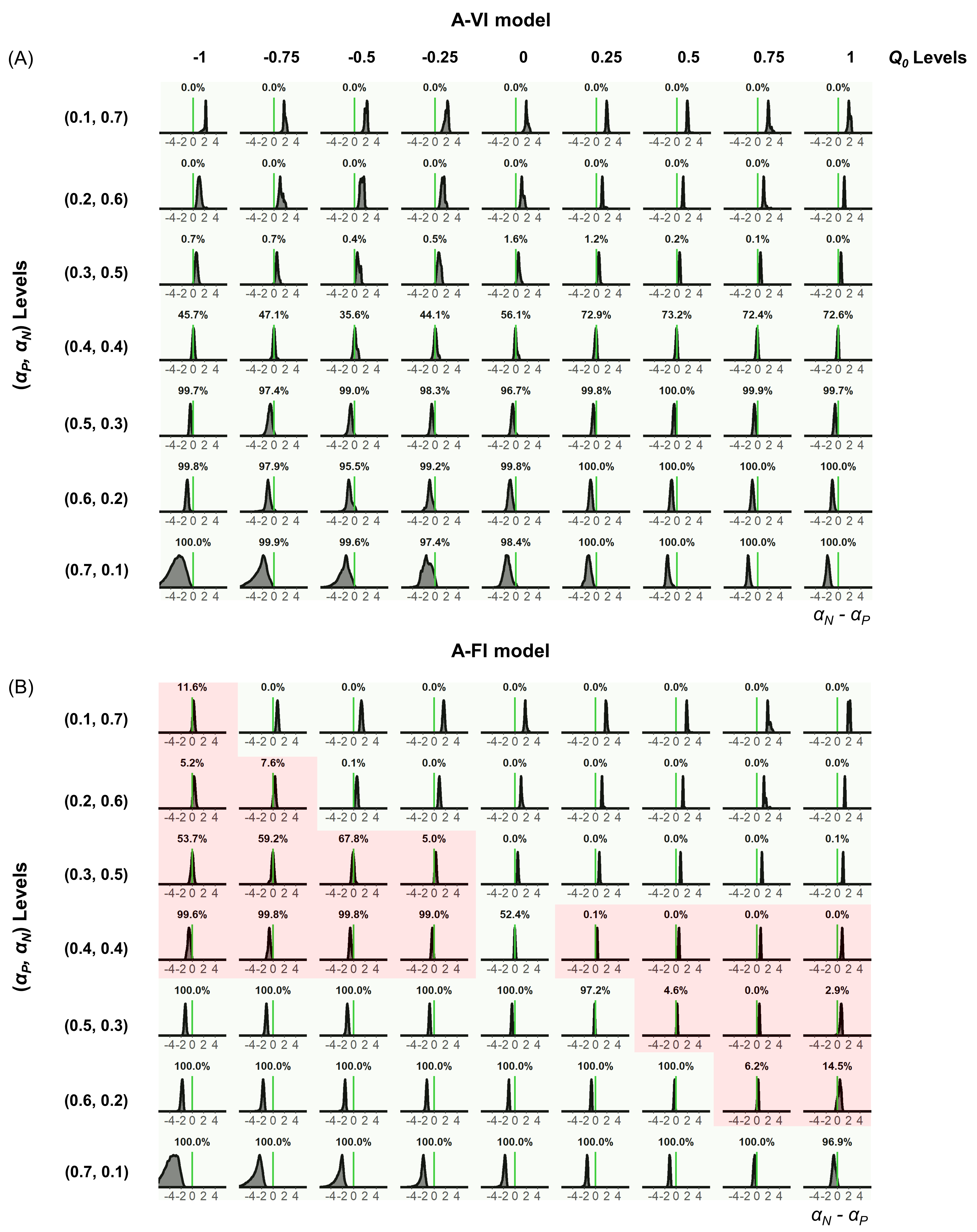

Supplement: S8 Fig — The posterior distribution of μδ, the hyper mean of the negative learning asymmetry for the A-VI model (A) and A-FI model (B). Light green in each distribution indicates the correct identification of learning asymmetry, whereas red shows the miscategorization for both models (A-VI and A-FI). (TIF) [file pcbi.1010751.s010.TIF]

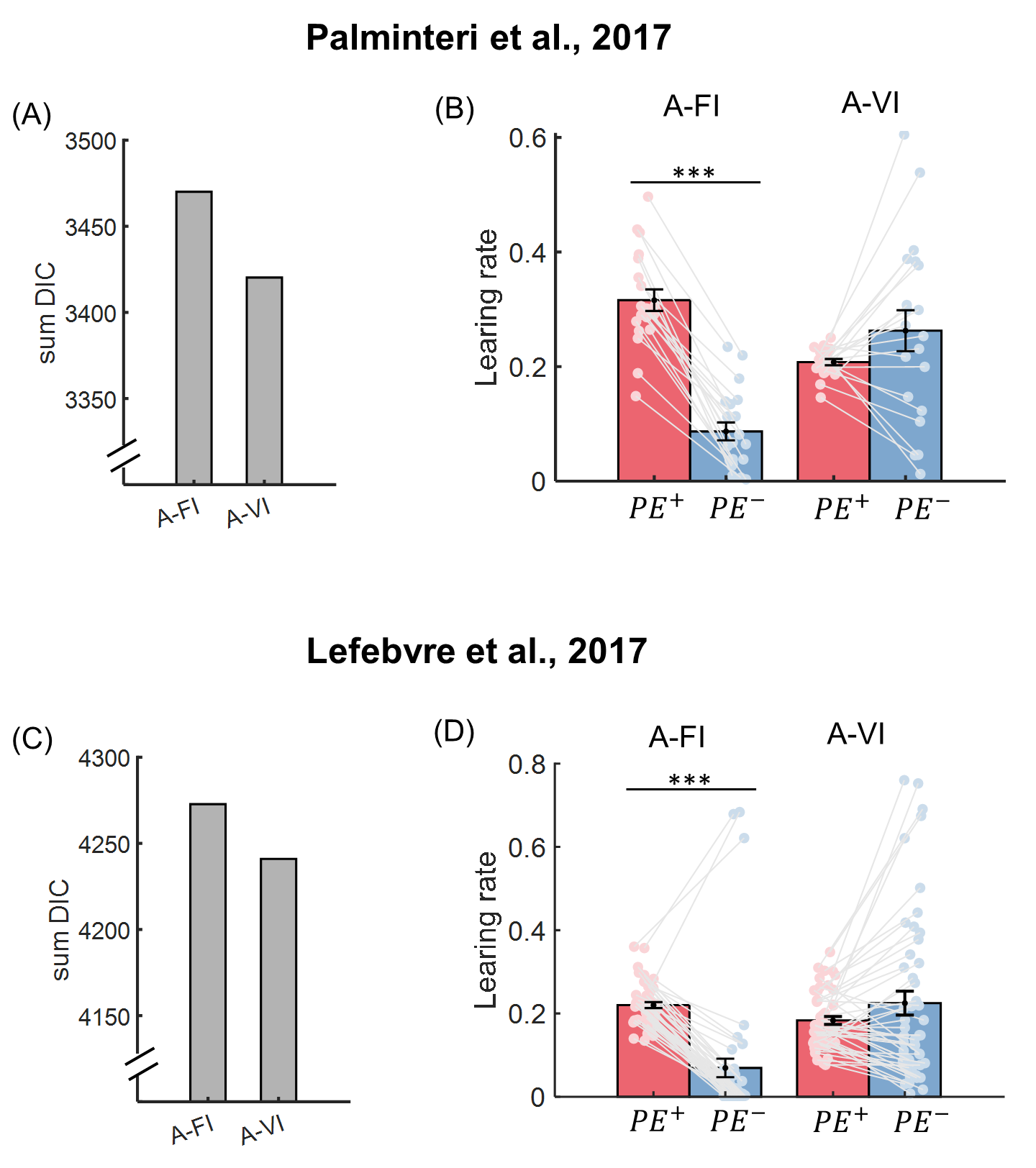

Supplement: S9 Fig — We selected publications in which the behavioral data were publicly available and the experimental designs were similar to ours and tested the consistency of our model performance [4,26]. The first dataset (N = 20) was from Palminteri et al.,2017 [28], in which four pairs of visual stimuli (24 trials per pair) were assigned with reward probabilities of 0.5/0.5, 0.75/0.25, 0.25/0.75 and 0.83/0.17). The second dataset (N = 50) was from Lefebvre et al.,2017 [4], where similar experimental paradigms were used except that the reward probabilities for the visual stimuli were slightly different (0.25/0.25, 0.75/0.75, 0.25/0.75 and 0.75/0.25, gain trials only). We fitted the behavioral data of the two datasets with two asymmetric learning rate models (A-VI and A-FI). The model fitting and comparison results suggest that the A-VI model performed better than the A-FI model (A and C). Without the initial value term, we found similar positive asymmetry biases as reported before (panels B and D). However, this pattern reversed when the initial value term was included in the A-VI model (interaction effect p-values < 0.001 for panels B and D). (TIF) [file pcbi.1010751.s011.TIF]
